# Wip1 inhibitor CCT007093 alleviates immune exhaustion of lymphocytes via p65 NF-κB and YY1 in chronic hepatitis B virus infection in mice

**DOI:** 10.3389/fimmu.2025.1548814

**Published:** 2025-05-09

**Authors:** Yu-Syuan You, Wan-Ting Chang, Chia-Lang Hsu, Hui-Ying Wang, Yan-Fong Lu, InKyeom Kim, Shiang-Jong Tzeng

**Affiliations:** ^1^ Graduate Institute of Pharmacology, College of Medicine, National Taiwan University, Taipei, Taiwan; ^2^ Department of Medical Research, National Taiwan University Hospital, Taipei, Taiwan; ^3^ Department of Obstetrics and Gynecology, Shin-Kong Wu Ho-Su Memorial Hospital, Taipei, Taiwan; ^4^ Department of Pharmacology, School of Medicine, Kyungpook National University, Daegu, Republic of Korea; ^5^ BK21 Plus KNU Biomedical Convergence Program, School of Medicine, Kyungpook National University, Daegu, Republic of Korea; ^6^ Cardiovascular Research Institute, School of Medicine, Kyungpook National University, Daegu, Republic of Korea

**Keywords:** CCT007093, p65 NF-kB, immune exhaustion, hepatitis B virus (HBV), FcγRIIB, PD-1, wild-type p53-induced phosphatase (Wip1), Ying Yang 1 (YY1)

## Abstract

**Introduction:**

Prolonged viral infections often lead to lymphocyte exhaustion, marked by heightened inhibitory receptor expression like PD-1, compromising host defense mechanisms. The unexplored potential of chemical checkpoint inhibitors in rejuvenating immune responses prompted our investigation.

**Methods:**

We focused on CCT007093, a Wip1 inhibitor, screened for its distinctive capacity to simultaneously decrease PD-1 and FcγRIIB expression in B cells.

**Results:**

In this study, we harnessed a murine model of immune exhaustion induced by chronic hepatitis B virus (HBV) infection using hydrodynamic injection. Treatment with CCT007093 resulted in decreased levels of PD-1 expression, resulting in reduced percentages of PD-1^+/hi^ CD4^+^ and CD8^+^ T cells in circulation, spleen, and liver. The expression levels of PD-1 and FcγRIIB, along with the percentages of PD-1^+/hi^ and FcγRIIB^+/hi^ CD19^+^ B cells in these tissues, were similarly diminished. Moreover, intrahepatic lymphocytes treated with CCT007093 displayed heightened responsiveness to *ex vivo* activation. Consequently, mice treated with CCT007093 exhibited significantly reduced serum HBsAg levels compared to vehicle-treated mice. Our detailed analyses, spanning promoter and transcriptome evaluations, uncovered p65 NF-κB as the primary activator of T cells and B cells, while Ying Yang 1 (YY1) emerged as the key regulator, orchestrating the down-regulation of PD-1 and FcγRIIB gene transcription in response to CCT007093.

**Discussion:**

Our study highlights the prowess of chemical checkpoint inhibitors, exemplified by CCT007093, in alleviating immune exhaustion in HBV-infected mice, particularly by enhancing adaptive immunity.

## Introduction

During chronic viral infections, the immune response can be hindered by immune exhaustion, characterized by the increased expression of inhibitory receptors on lymphocytes, such as cytotoxic T-lymphocyte-associated protein 4 (CTLA4), programmed death-1 (PD-1), and programmed death-ligand 1 (PD-L1). This exhaustion negatively affects the activation, proliferation, and differentiation of lymphocytes (1, 2). In chronic HBV infection, both CD4^+^ and CD8^+^ T cells show elevated levels of PD-1, indicating T cell exhaustion (3, 4). Similarly, B cells in HIV-infected patients exhibit exhaustion with increased expression of FcγRIIB, leading to impaired B cell function and antibody (Ab) production (5). Taken together, T-cell exhaustion weakens the cytotoxic response necessary for clearing the virus, while B-cell exhaustion compromises the effectiveness of the humoral response against the virus. However, the factors that can ameliorate or reverse lymphocyte exhaustion in order to promote activation of T cells and B cells in chronic viral infections for the treatment of patients require further research.

Recovering from immune exhaustion in persistent viral infections represents a primary objective in the advancement of novel therapeutics. Research has generated compelling evidence endorsing the targeting of inhibitory receptors with checkpoint inhibitors, such as the use of monoclonal Abs aimed at PD-1, which has proven effective in enhancing T cell functionality in individuals with human HIV and HBV infections (6, 7). Additionally, the remarkable success of anti-PD-1 and anti-PD-L1 monoclonal Abs in cancer therapy underscores the potential importance of targeting these pathways for the prevention and management of various infectious diseases (8–10). While antiviral drugs continue to be the mainstay treatment for immune exhaustion and chronic viral infections, immune checkpoint inhibitors like anti-PD-1 have emerged as potential therapeutic options. However, the effectiveness of antiviral drugs in combating immune exhaustion remains uncertain. Additionally, the widespread use of checkpoint inhibitors is hindered by factors such as high cost, long circulating half-life, and the potential for severe side effects, including life-threatening cytokine storms. Therefore, further research and development of safer and more cost-effective therapies are necessary to address immune exhaustion and chronic viral infections effectively.

Chemical compounds offer an alternative approach to deliver immune checkpoint inhibition, addressing some of the challenges associated with monoclonal Ab inhibitors. Chemical compounds generally have a faster turnover rate than monoclonal Abs in circulation, making them potentially more suitable for therapeutic applications. Furthermore, the risk of cytokine storms may be reduced compared to monoclonal Ab-based therapies. Importantly, they can be manufactured more easily and at a lower cost, allowing for broader accessibility for patients. Therefore, the development of chemical compounds as immune checkpoint inhibitors holds promise for overcoming the limitations of monoclonal Abs and improving the treatment of immune exhaustion and chronic viral infections.

By employing a cell-based gene promoter reporter assay, we conducted a screen of compounds with the capability to reduce the gene transcription of both FcγRIIB and PD-1 in BJAB B cells (11). From the pool of candidate compounds identified, our focus narrowed to CCT007093, an inhibitor of wild-type p53-induced phosphatase 1 (Wip1, also known as PPM1D). Wip1 is a serine/threonine phosphatase mainly localized in the nucleus (12). To assess the potential of CCT007093 in countering immune exhaustion within the context of chronic viral infection, we harnessed an established murine model of chronic hepatitis B virus (HBV) infection for therapeutic and mechanistic investigations (13).

## Methods

### Reagents

CCT007093 (IC_50_: 8.4 μM) and DMSO were purchased from Selleckchem (Houston, TX, USA). Recombinant HBsAg and HBcAg proteins were acquired from Sino Biological. For immunoblotting, specific Abs to detect p65 NF-κB, phospho-p65 NF-κB (Ser 536), p53, phospho-p53 (Ser 15), and Wip1 were purchased from Cell Signaling Technology (Beverly, MA, USA). Anti-PD-1 and anti-YY1 Abs were purchased from Santa Cruz (Santa Cruz, CA, USA), while anti-FcγRIIB Ab was ordered from Genetex Inc. (Irvin, CA, USA). Cell activation kit (ionomycin + PMA (phorbol-12-myristate-13-acetate)) and anti-IFN-γ Abs were purchased from Biolegend. Anti-β-actin Ab was ordered from Novus Biologicals (Littleton, CO, USA). Anti-myc Ab was purchased from Proteintech (Rosemont, IL, USA). The protein G-sepharose beads were sourced from Sigma-Aldrich (St. Louis, MO, USA). Both horseradish peroxidase-conjugated goat anti-rabbit and anti-mouse Abs were obtained from Jackson ImmunoReasearch (West Grove, PA, USA). Monoclonal Abs for flow cytometry were ordered from Biolegend and BD Biosciences (Becton, NJ, USA).

### Plasmids

The HBc175 mutant pAAV/HBV1.2 construct was generously provided by Dr. Pei-Jer Chen at National Taiwan University. This construct expresses HBcAg proteins with a C-terminal deletion of 10 amino acids (13). The Wip1 cDNA plasmid (GV227-Wip1) was obtained from GeneChem (Shanghai, China) and subsequently subcloned into the pcDNA3.1/myc-His B vector. The pCMV6-XL5-YY1 plasmid was acquired from Origene (Rockville, MD, USA). Serum HBsAg levels were measured with an AXSYM system kit (Abbott GmbH Diagnostica, Wiesbaden, Germany).

### Animals and hydrodynamic injection

Male C57BL/6J mice, aged 5 to 6 weeks, were acquired from the Center for Laboratory Animals of the College of Medicine, National Taiwan University (NTU). These mice were housed under specific pathogen-free conditions. All animal experiments were conducted in accordance with ethical guidelines and approved protocols from the Institutional Animal Care and Use Committee (IACUC) of the College of Medicine, NTU, under the protocol numbers: 20130228 and 20170401. The hydrodynamic injection procedure, aimed at inducing HBV-associated immune exhaustion in mice, has been previously documented (13, 14). In brief, we administered 10 μg of purified HBc175 plasmid DNAs in approximately 1.5 ml PBS (equivalent to 8% of each mouse’s body weight) via the tail vein, resulting in rapid hepatocyte rupture for *in vivo* transfection. Starting four weeks after the hydrodynamic injection, serum levels of HBsAg in mice were monitored at 2-week intervals using an AXSYM system kit (Abbott GmbH Diagnostica, Wiesbaden, Germany). The persistence of HBsAg in the serum beyond four weeks post-injection indicates the successful induction of chronic HBV infection and immune exhaustion. This method enables HBV persistence for over a year in a substantial proportion of mice (8, 13). Typically, we achieved successful immune exhaustion in approximately 50-60% of the mice.

### Cell preparation from blood, spleen and liver

Mouse blood was collected by puncturing the submandibular vein using lancets, with the blood being collected into a K_2_EDTA-coated tube (BD Biosciences). Splenocytes were isolated as previously detailed (15–18). To isolate intrahepatic leukocytes, the mouse liver was minced using scissors. Sections of the liver were placed into two 10-cm petri dishes, each containing 10 ml of digestion buffer (0.04% collagenase IV and 0.002% DNase I dissolved in PBS). The liver pieces were gently crushed with a sterile syringe plunger and passed through a 100-μm cell strainer to eliminate connective tissues. The liver suspensions were then incubated at 37°C for 45 min in an incubator. Subsequent to incubation, the liver cell mixture was collected into a 50 ml conical tube and centrifuged at 30×g (500 rpm) for 3 min to separate hepatocytes. Following centrifugation, the cell pellets were re-suspended in RBC lysis buffer and incubated at room temperature for 5 min to eliminate erythrocytes. This was followed by another centrifugation at 2,200 rpm for 15 min. The pellets were then re-suspended in 4 ml of PBS and cautiously layered onto the top of a Percoll gradient, containing 4 ml of 25% Percoll solution (GE Healthcare, Piscataway, NJ). The layer of 25% Percoll was placed over the 4 ml of 80% Percoll in a 15 ml tube. The cells were then centrifuged at 2,500 rpm at 25°C for 25 min. The intrahepatic leukocytes were carefully collected from the interface between the 25% and 80% Percoll layers and subsequently washed with 10 ml of PBS. Following a centrifugation step at 2,200 rpm for 10 min, the resulting cell pellets were harvested for subsequent flow cytometric analysis. Typically, we isolated 1-2 × 10^6^ leukocytes from the liver of each mouse.

### Flow cytometry

For T cells, specific Abs of CD45-BV421 (clone 104), CD3-APC-Cy7 (clone 145-2C11), CD4-Alexa 647 (clone GK1.5), CD8-BV510 (clone 53-6.7), PD-1-PE (clone 29F.1A12), and CD127-FITC (clone A7R34) were added and incubated at 4°C for 20 min. 7-AAD was used for exclusion of dead cells. For additional analysis of T cells, Tim-3-PE (clone B8.2C12), CTLA-4-PE (clone UC10-4B9), TIGIT-PE (clone 4D4/mTIGIT) were used, replacing PD-1-PE in the staining protocol. For B cells, the CD16/32-FITC Abs (clone 2.4G2) were initially added for a 5-min incubation period, followed by an additional 20-min incubation with CD19-PE-Cy7 (clone 6D5) and PD-1-PE Abs at 4°C. For hepatic Kupffer cells, CD11b-FITC (clone M1/70), F4/80-PerCP-Cy5.5 (clone BM8) and PD-L1-PE (clone 10F.9G2) were used. For intracellular staining, isolated intrahepatic leukocytes (5×10^6^/ml) were cultured in the presence of a stimulation agent cocktail for a duration of 3 hr. Subsequently, the cells were collected and labeled with CD3-APC-Cy7 (clone 145-2C11), CD4-PerCP-Cy5.5 (clone RM 4-5), and CD8-BB515 (clone 53-6.7) Abs on ice for a period of 20 min. After the completion of the staining process, the cells were subjected to permeabilization and fixation using a fixation/permeabilization kit (BD Biosciences, #555028) following the manufacturer’s instruction. This was followed by the addition of IFN-γ-PE (clone XMG1.2) or rat IgG1-PE isotype (clone G0114F7) Abs, with an additional 30-min incubation on ice. After the necessary washing steps, the cells were analyzed using flow cytometry. Fluorescence minus one (FMO) controls were included in the analysis for detecting inhibitory receptors and IFN-γ expression. Extending from previously described protocols (15–17), the gating strategies for T cells and B cells in circulation, spleen, and liver are highlighted in **Supplementary Figures 1**-**3**, respectively. To quantify IFN-γ-expressing T cells in the liver, the corresponding gating strategies are shown in **Supplementary Figure 4**.

### Enzyme-linked immunospot

ELISPOT plates (BD Biosciences) were coated overnight at 4°C with recombinant HBsAg or HBcAg proteins (Sino Biological) at a concentration of 2 μg/ml. Approximately 10^5^ purified intrahepatic leukocytes was used for the ELISPOT assay, and the experiments were conducted following previously described protocols (17).

### Cell culture and dual-luciferase reporter assay

BJAB B cells is a Epstein-Barr virus-negative Burkitt-like lymphoma cell line (17). Jurkat T cells and HEK293T cells were grown in RPMI 1640 medium supplemented with 10% fetal bovine serum and penicillin/streptomycin/Amphotericin B, and maintained in an incubator with 5% of CO_2_ at 37°C. The dual-luciferase reporter system from Promega (Madison, WI, USA) was utilized to measure the transcriptional activities of promoters. For cell transfection, the Amaxa™ Nucleofector kit V from Lonza (Basel, Switzerland) was used. Briefly, 1×10^7^ BJAB B cells were transfected with 20 μg of pGL3 luciferase reporter plasmids containing 2,200 bp, 1,636 bp, 1,075 bp and 926 bp promoter sequences upstream of ATG of PD-1 gene (Genebank access no. AF363458), respectively. Simultaneously, the *renilla* luciferase reporter constructs (500 ng) was co-transfected as internal controls for transfection efficiency and for normalization of luciferase activities. Following cell electroporation for 4 hr, cells were treated with vehicle, 2 or 5 μM CCT007093 in triplicates and incubated for an additional 24 hr. Cells were lysed, and luciferase activities were measured using the dual-luciferase reporter kit (Promega) following the manufacturer’s instructions. Luciferase activities were presented as the ratio of firefly to *renilla* luciferase activities (Fluc/Rluc). Similarly, the 1.5 kb and 0.6 kb promoter sequences of FcγRIIB gene (Genebank access no. AF433951) were linked to firefly luciferase constructs for similar analysis.

### Western blotting and immunoprecipitation

The western blotting procedures followed standard methods. BJAB B cells were lysed in RIPA buffer containing protease and phosphatase inhibitor cocktails on ice for 30 min, followed by a brief sonication. Cell lysates were then centrifuged at 12,000×g at 4°C for 15 min, and the resulting supernatant was transferred into a new Eppendorf tube. Protein concentration was determined using the Coomassie blue assay kit (Thermo Fisher) according to the manufacturer’s instructions. Proteins in lysates were separated by SDS-polyacrylamide gel electrophoresis (PAGE; 8%-10%). After the gel transfer, membranes were blocked with TBS-T buffer (50 mM Tris-HCl, pH 7.6, 150 mM NaCl and 0.1% Tween 20) containing 5% non-fat milk at ambient temperature for 1 hr to reduce non-specific protein binding. Primary Abs were added and incubated overnight at 4°C. Following three washes with TBS-T, HRP-conjugated secondary Abs (Jackson ImmunoResearch) were added and incubated at ambient temperature for 1 hr. After completing incubation, membranes were washed six times with TBS-T. Protein bands specific to the Abs were detected using a chemiluminescence ECL kit. The β-actin was used as an internal control.

HEK 293T cells (2.4×10^5^/ml) were seeded in 6-well plates and allowed to grow for 24 hr prior to transfection. For transfection, the culture medium in each well was replaced with a mixture of full and serum-free media at a ratio of 1:1 (200 μl), along with 4 μg of each plasmid in 8 μl of TurboFect transfection reagent (Thermo Fisher Scientific). After incubation at 37°C for 24 hr, the cells were harvested for western blotting analysis as described earlier. For immunoprecipitation, protein G-sepharose beads (20 μl) were added to each sample (500 μg) and incubated overnight at 4°C. The Ab-specific immune complexes were then washed with cold lysis buffer and TBS-T containing protease and phosphatase inhibitors. After boiling in the sample loading buffer for 10 min, the samples were resolved in 8%-10% SDS-PAGE gels for subsequent western blotting.

### RNA extraction and sequencing of splenic B cells

Total RNAs of Jurkat T cells and BJAB B cells were extracted for RNA-sequencing following the established protocol (16, 19). RNA was extracted using Trizol. The purified RNA was utilized for library preparation using the TruSeq stranded mRNA library prep kit (Illumina, San Diego, CA, USA), following the manufacturer’s instructions. Briefly, mRNA was purified from 1 µg of total RNA using oligo(dT)-coupled magnetic beads and fragmented into smaller fragments. The first-strand cDNA was synthesized using reverse transcriptase and random primers. Double-stranded cDNA was generated, and the DNA fragments’ 3’ ends were adenylation-modified. Adaptors were ligated to the fragments, which were subsequently purified using the AMPure XP system (Beckman Coulter, Beverly, USA). The quality of the libraries was assessed using the Agilent Bioanalyzer 2100 system and real-time PCR. qualified libraries were sequenced on an Illumina NovaSeq 6000 platform, generating 150 bp paired-end reads, by Genomics, BioSci & Tech Co., New Taipei City, Taiwan.

### Bioinformatic analysis

The raw reads underwent quality checking using FastQC (v0.11.9), and adaptor sequences as well as low-quality reads were eliminated using cutadapt (v3.5). The analysis of qualified reads, aligned to the mouse reference genome GRCh38 using STAR (v2.7.8a) with the two-pass mode, was performed as previously described (19). Gene-level read counts were calculated based on the Gencode v35 annotation. Cross-sample normalization was conducted using the TMM (Trimmed Mean of the M-values) method implemented in the R package edgeR, and transcript per million (TPM) values were calculated for further analysis. Differential expression analysis was performed using the R package NOISeq, and genes with a probability greater than or equal to 0.4 were considered as differentially expressed genes. Pre-rank gene-set enrichment analysis (GSEA) was carried out using the functions of the R package clusterProfiler on gene-sets sourced from MSigDB (v7.4). Genes were ranked based on the rank * probability, where the rank and probability values were obtained from the NOISeq results. Pathway enrichment and functional associated networks were constructed using the Ingenuity Pathway Analysis (IPA).

### Statistical analysis

FlowJo software and Student’s *t*-test was used to analyze flow cytometric data. Plots were generated using GraphPad Prism 7.0 (GraphPad Software Inc., USA) and the results were presented as mean ± standard error (SEM). P-values below 0.05 are considered statistically significant between compared groups and denoted by asterisks: *P < 0.05, **P < 0.01, and ***P < 0.001.

## Results

### CCT007093 treatment reduced serum HBsAg levels in chronic HBV-infected mice

**Figure 1:**
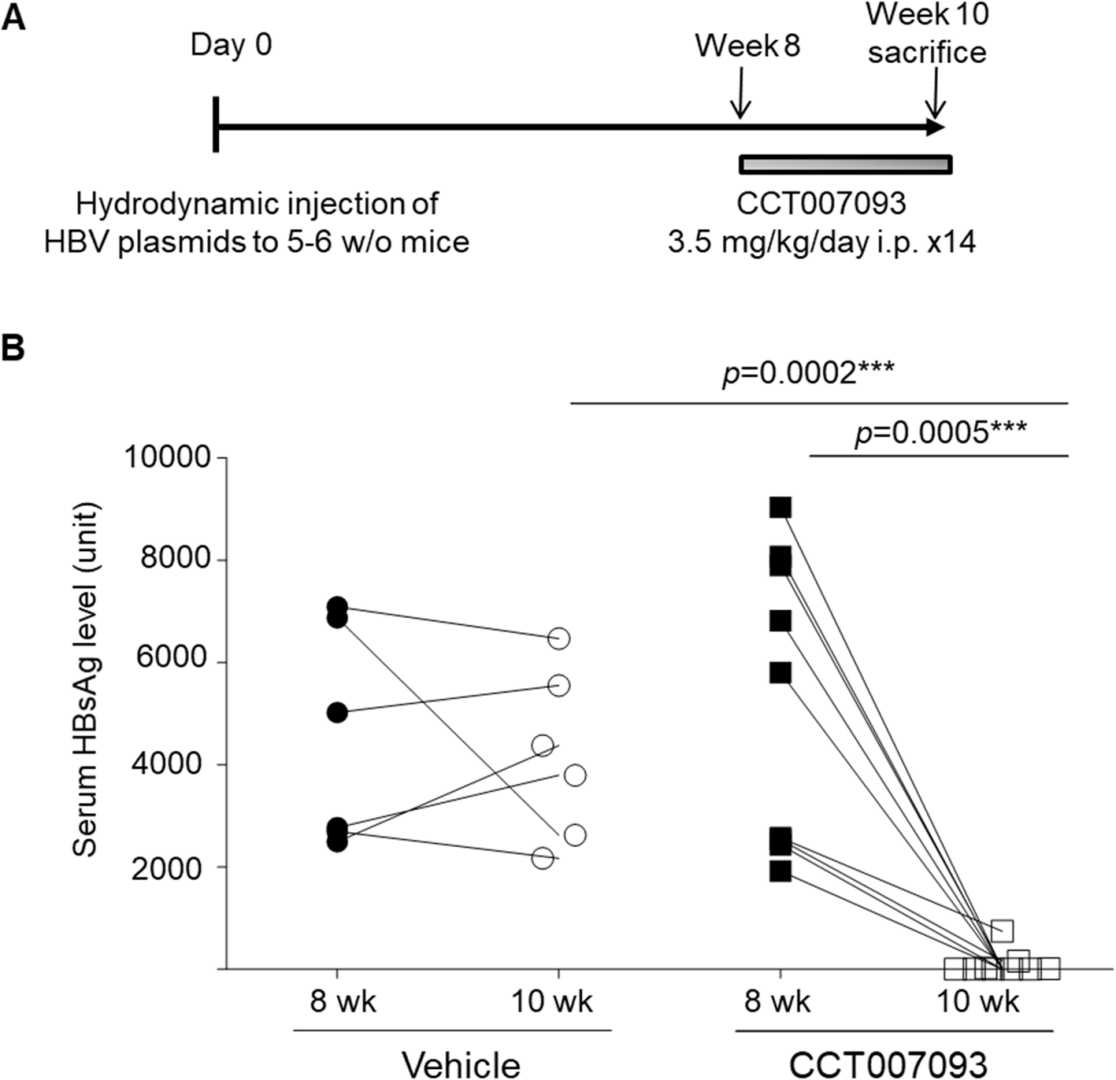
CCT007093 treatment markedly reduced serum HBsAg levels in chronic HBV-infected mice. **(A)** Schematic illustration delineating the induction of immune exhaustion through chronic HBV infection (detailed in Methods) and the treatment protocols involving either vehicle (PBS, represented by circles, n=6) or CCT007093 (displayed as squares, n=8). **(B)** Male mice underwent an 8-week protocol to confirm the successful induction of immune exhaustion before treatment initiation. Serum HBsAg levels were measured before and after a 2-week treatment period using either vehicle or CCT007093. Mouse serum samples were diluted threefold for HBsAg quantification. ***P < 0.001.

We utilized a well-established murine model of chronic HBV infection characterized by T-cell exhaustion to evaluate the therapeutic potential of CCT007093. We chose male C57BL/6 mice due to their higher susceptibility to HBV infection, mirroring the clinical scenario (13, 20). Chronic HBV infection was induced via hydrodynamic injection of HBc175 plasmids, leading to immune exhaustion. Eight weeks post-infection, mice were treated with either vehicle control or CCT007093 at a dose of 3.5 mg/kg/day for 14 days (**Figure 1A**). T-cell exhaustion was confirmed by increased PD-1 expression on T cells and the sustained presence of serum HBsAg as previously described (8). At the conclusion of the study, mice were sacrificed to assess immune restoration based on cellular phenotypes and functional responses. As shown in **Figure 1B**, mice infected with HBV and treated with CCT007093 exhibited a notable decrease in serum HBsAg levels compared to vehicle-treated controls, underscoring its therapeutic potential in alleviating immune exhaustion and promoting viral clearance.

### CCT007093 downregulated PD-1 and FcγRIIB expression in lymphocytes in blood, spleen and liver of chronic HBV-infected mice

**Figure 2:**
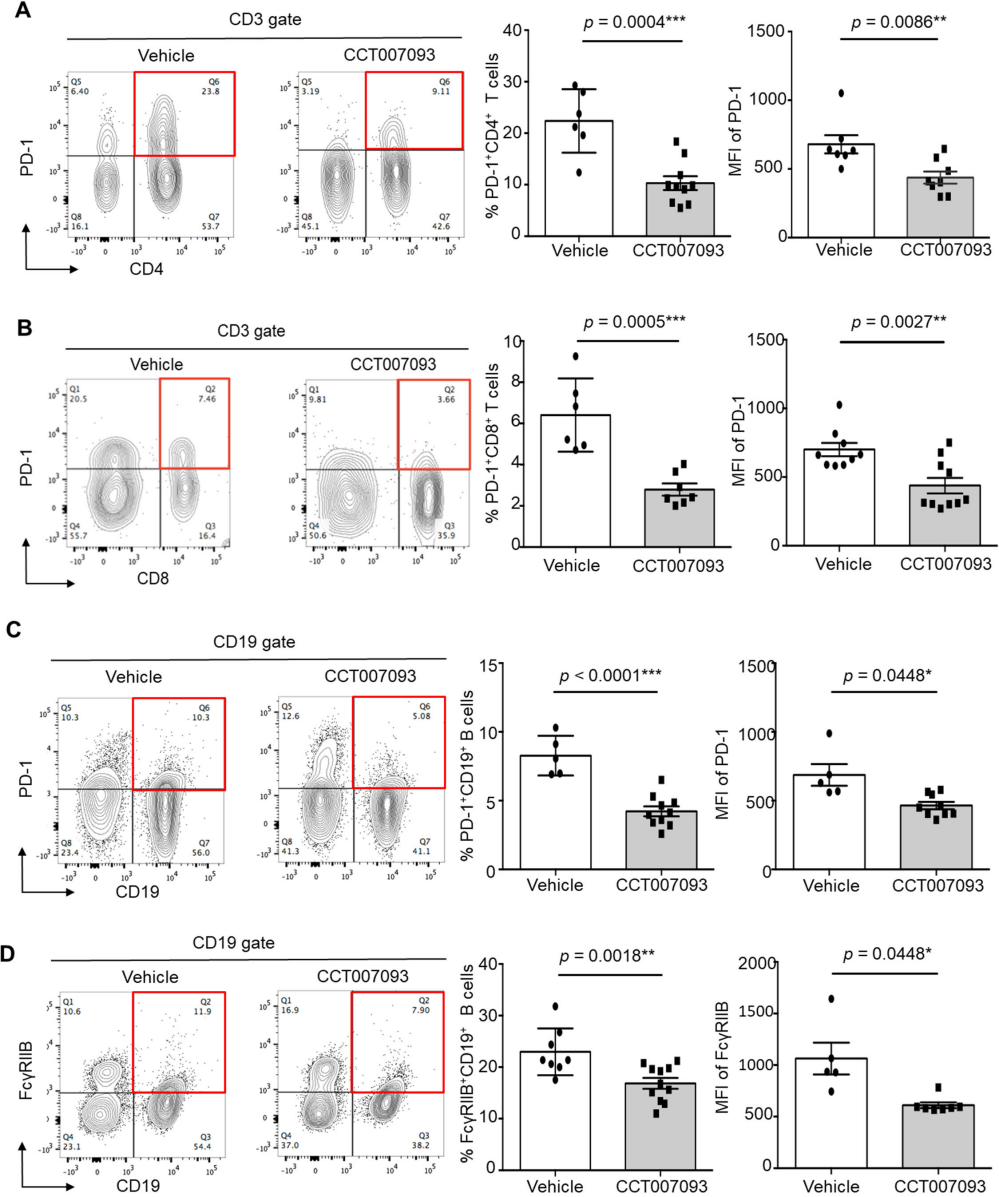
CCT007093 attenuated PD-1 and FcγRIIB expression in circulating lymphocytes of chronic HBV-infected mice. **(A)** Flow cytometry data (left) illustrating PD-1^+/hi^CD4^+^ T cells in peripheral blood from immune exhausted HBV-infected mice treated with either vehicle or CCT007093. The middle section displays the percentages of PD-1^+/hi^CD4^+^ T cells, and the right section indicates mean fluorescence intensity (MFI) of PD-1 expression levels in these T cells for mice treated with vehicle (solid circles in white bar, n=6) or CCT007093 (solid squares in grey bar, n=8-9). Data were analyzed using FlowJo software. **(B)** Flow cytometry analysis (left) demonstrating circulating PD-1^+/hi^CD8^+^ T cells from HBV-infected mice with immune exhausted treated with either vehicle or CCT007093. The middle panel indicates the percentages of PD-1^+/hi^CD8^+^ T cells, while the right panel illustrates the MFI of PD-1 expression levels in these T cells for mice treated with vehicle (solid circles, n=6) or CCT007093 (solid squares, n=7). **(C)** Flow cytometry data (left) showing circulating PD-1^+/hi^CD19^+^ B cells from HBV-infected mice with immune exhaustion treated with either vehicle or CCT007093. The middle section represents the percentages of PD-1^+/hi^CD19^+^ B cells, and the right section exhibits the MFI of PD-1 expression levels for mice treated with vehicle (solid circles, n=5) or CCT007093 (solid squares, n=9). **(D)** Flow cytometry analysis (left) illustrating circulating FcγRIIB^+/hi^CD19^+^ B cells from HBV-infected mice with immune exhaustion treated with either vehicle or CCT007093. The middle section shows the percentages of FcγRIIB^+/hi^CD19^+^ B cells, while the right section demonstrates the MFI of FcγRIIB expression levels in these B cells for mice treated with vehicle (solid circles, n=5) or CCT007093 (solid squares, n=9). All data were analyzed using FlowJo software. The statistical significance was determined using the two-tailed unpaired *t*-test, and significance levels are indicated as follows: *P < 0.05, **P < 0.01, ***P < 0.001.

We then conducted a flow cytometric analysis to assess the surface expression levels of PD-1 and FcγRIIB on both CD4^+^ and CD8^+^ T cells, as well as B cells, in the peripheral blood, spleen, and liver using flow cytometry. Our results revealed a significant reduction in PD-1 surface expression on circulating CD4^+^ (**Figure 2A**) and CD8^+^ (**Figure 2B**) T cells, accompanied by a decreased percentages of PD-1^+/hi^ CD4^+^ and CD8^+^ T cells in mice treated with CCT007093. Given that exhausted human T cells typically exhibit elevated expression of multiple inhibitory receptors (2–4), we further examined the expression levels of Tim-3, CTLA-4, and TIGIT on circulating CD4^+^ and CD8^+^ T cells. However, no significant differences were observed between the vehicle- and CCT007093-treated groups (**Supplementary Figure 1**). Next, we investigated the status of exhausted B cells in the peripheral blood and found that CCT007093 treatment led to decreased surface expression of both PD-1 (**Figure 2C**) and FcγRIIB (**Figure 2D**). Additionally, the percentages of PD-1^+/hi^ and FcγRIIB^+/hi^ CD19^+^ B cells were significantly reduced in CCT007093-treated mice (**Figures 2C, D**).

**Figure 3:**
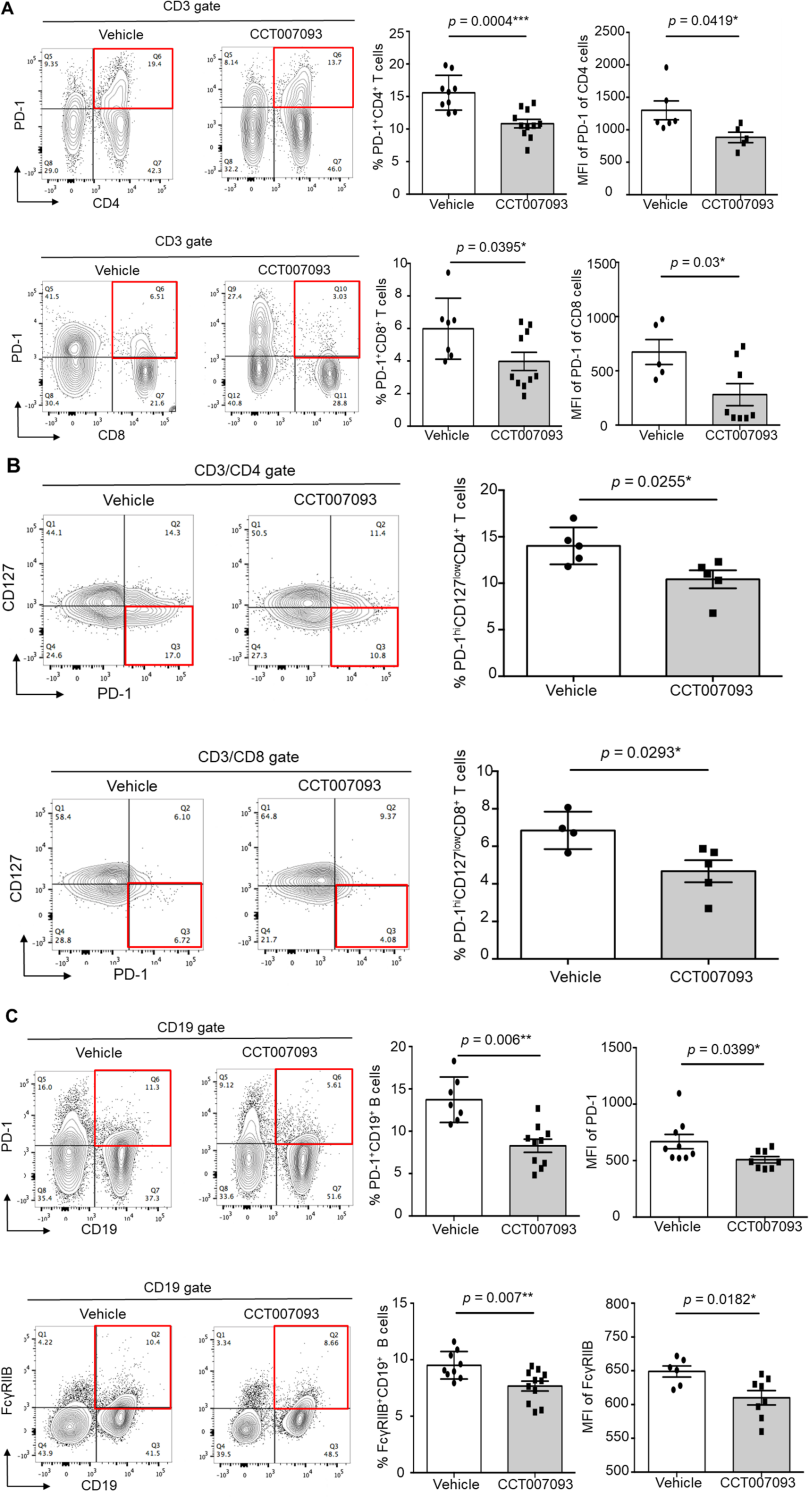
CCT007093 reduced PD-1 and FcγRIIB expression in splenic lymphocytes of chronic HBV-infected mice. **(A)** Flow cytometric analysis illustrating PD-1^+/hi^CD4^+^ (left upper) and PD-1^+/hi^CD8^+^ T cells (left lower) in splenocytes from immune exhausted HBV-infected mice treated with either vehicle or CCT007093. Quantification results showed the percentages of PD-1^+/hi^CD4^+^ (upper middle) and PD-1^+/hi^CD8^+^ (lower middle) T cells, as well as the MFI of PD-1 expression levels for PD-1^+/hi^CD4^+^ (upper right) and PD-1^+/hi^CD8^+^ (lower right) T cells in mice treated with vehicle (solid circles in white bar, n=5-8) or CCT007093 (solid squares in grey bar, n=5-10), respectively. **(B)** Flow cytometric analysis depicting PD-1^+/hi^CD127^lo^CD4^+^ (left upper) and PD-1^+/hi^CD127^lo^CD8^+^ T cells (left lower) in splenocytes from immune exhausted HBV-infected mice treated with either vehicle or CCT007093. Quantification results showed the percentages of PD-1^+/hi^CD127^lo^CD4^+^ (upper middle) and PD-1^+/hi^CD127^lo^CD8^+^ (lower middle) T cells, along with the MFI of PD-1 expression levels for PD-1^+/hi^CD127^lo^CD4^+^ (right upper) and PD-1^+/hi^CD127^lo^CD8^+^ (right lower) T cells in mice treated with vehicle (solid circles, n=4-5) or CCT007093 (solid squares, n=5). **(C)** Flow cytometric analysis representing PD-1^+/hi^CD19^+^ (left upper) and FcγRIIB^+/hi^CD19^+^ B cells (left lower) in splenocytes from immune exhausted HBV-infected mice treated with either vehicle or CCT007093. Quantification results disclosed the percentages of PD-1^+/hi^CD19^+^ (upper middle) and FcγRIIB^+/hi^CD19^+^ (lower middle) B cells, as well as the MFI of PD-1 expression levels for PD-1^+/hi^CD19^+^ (upper right) and FcγRIIB^+/hi^CD19^+^ (lower right) B cells in mice treated with vehicle (solid circles, n=6-9) or CCT007093 (solid squares, n=8-11). Statistical significance was determined using a two-tailed unpaired *t*-test, with significance levels indicated as follows: *P < 0.05, **P < 0.01, ***P < 0.001.

When analyzing splenic leukocytes, we observed a significant reduction in PD-1 expression levels on CD4^+^ and CD8^+^ T cells, as quantified by MFI, as well as a decreased percentage of PD-1^+/hi^ CD4^+^ and CD8^+^ T cells in CCT007093-treated mice (**Figure 3A**). Notably, recent studies have indicated that HBV-specific dysfunctional T cells exhibit high PD-1 levels coupled with low CD127 (also known as IL-7 receptor) expression in both chronic HBV patients and mice (8, 21). To further investigate this, we compared the frequency of exhausted splenic T cells, defined as PD-1^+/hi^CD127^lo^CD4^+^ and PD-1^+/hi^CD127^lo^CD8^+^ T cells. Following CCT007093 treatment, the percentages of these exhausted T cell populations were significantly reduced in comparison to those in the vehicle-treated mice (**Figure 3B**). Additionally, we assessed the expression levels of other inhibitory receptors, including Tim-3, CTLA-4, and TIGIT, on splenic CD4^+^ and CD8^+^ T cells; however, no significant differences were observed between the treatment groups (**Supplementary Figure 6**). In contrast, CCT007093 treatment led to a marked decrease in both the expression levels and percentages of PD-1^+/hi^ B cells and FcγRIIB^+/hi^ B cells in the spleen compared to vehicle-treated mice (**Figure 3C**).

**Figure 4:**
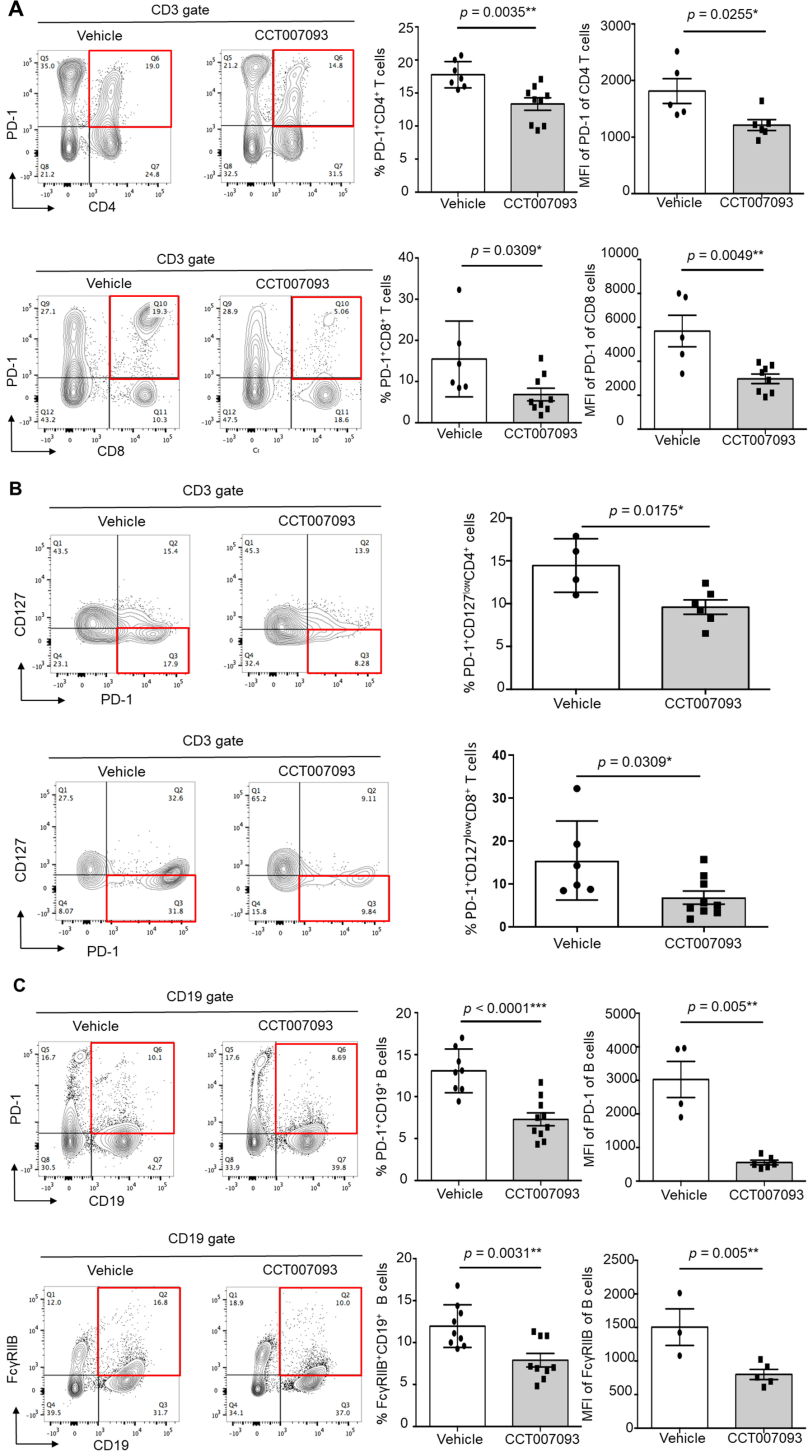
CCT007093 downregulated PD-1 and FcγRIIB expression levels in intrahepatic lymphocytes of immune exhausted HBV-infected mice. **(A)** Representative flow cytometric analysis depicting the expression levels of PD-1 in intrahepatic CD4 T cells (upper left) and CD8 T cells (lower left) in HBV-infected mice treated with vehicle or CCT007093. The middle panels displayed quantification results of CD4 (upper) and CD8 (lower) T cells in bar graphs (vehicle in white bar; CCT007093 in grey bar), while the right panels illustrated the percentages of PD-1^+/hi^CD4^+^ (upper, n=5-9) and PD-1^+/hi^CD8^+^ (lower, n=5-9) intrahepatic T cells, respectively. **(B)** Flow cytometric analysis (left) and quantification (right) showing the percentages of PD-1^+/hi^CD127^low^CD4^+^ (upper, n=4-6) and PD-1^+/hi^CD127^low^CD8^+^ T cells (lower, n=4) in HBV-infected mice treated with vehicle or CCT007093. **(C)** Flow cytometric analysis (left) of the expression levels of PD-1 (upper) and FcγRIIB (lower) in intrahepatic CD19^+^ B cells in HBV-infected mice treated with vehicle or CCT007093. The middle panels exhibited quantification results of PD-1^+^ (upper) and FcγRIIB^+^ (lower) B cells in bar graphs, while the right panels illustrated the percentages of intrahepatic PD-1^+/hi^ (upper) and FcγRIIB^+/hi^ CD19^+^ B cells, respectively, in HBV-infected mice treated with vehicle (solid circles, n=3-8) or CCT007093 (solid squares, n=5-10). Statistical significance was determined using a two-tailed unpaired *t*-test, with significance levels indicated as follows: *P < 0.05, **P < 0.01, ***P < 0.001.

To assess the localized therapeutic effects of CCT007093 within the liver, we analyzed intrahepatic leukocytes from HBV-infected mice. As shown in **Figure 4**, both the expression levels and percentages of PD-1^+/hi^ CD4^+^ T cells and CD8^+^ T cells were markedly reduced in CCT007093-treated mice in comparison to vehicle-treated mice. Consistent with our splenic findings (**Figure 3**), the percentages of intrahepatic PD-1^+/hi^CD127^lo^CD4^+^ and PD-1^+/hi^CD127^lo^CD8^+^ T cells were significantly lower in CCT007093-treated mice (**Figures 4A, B**). Additionally, the expression levels and percentages of PD-1^+/hi^ B cells and FcγRIIB^+/hi^ B cells were substantially reduced in CCT007093-treated mice than in vehicle-treated mice (**Figure 4C**). However, similar to our observations in circulating and splenic T cells, the expression levels of Tim-3, CTLA-4, and TIGIT expression on intrahepatic CD4^+^ and CD8^+^ T cells remained unchanged between treatment groups (**Supplementary Figure 7**). To further investigate liver-resident leukocytes, we analyzed the percentages and expression levels of PD-L1 on Kupffer cells (CD45^+^CD3^-^CD11b^+^F4/80^+/hi^). Notably, PD-L1 expression was significantly reduced following CCT007093 treatment, accompanied by an increased number of Kupffer cells (**Supplementary Figure 8**).

### CCT007093 treatment enhanced IFN-γ and antiviral Ab production by intrahepatic lymphocytes in HBV-infected mice

**Figure 5:**
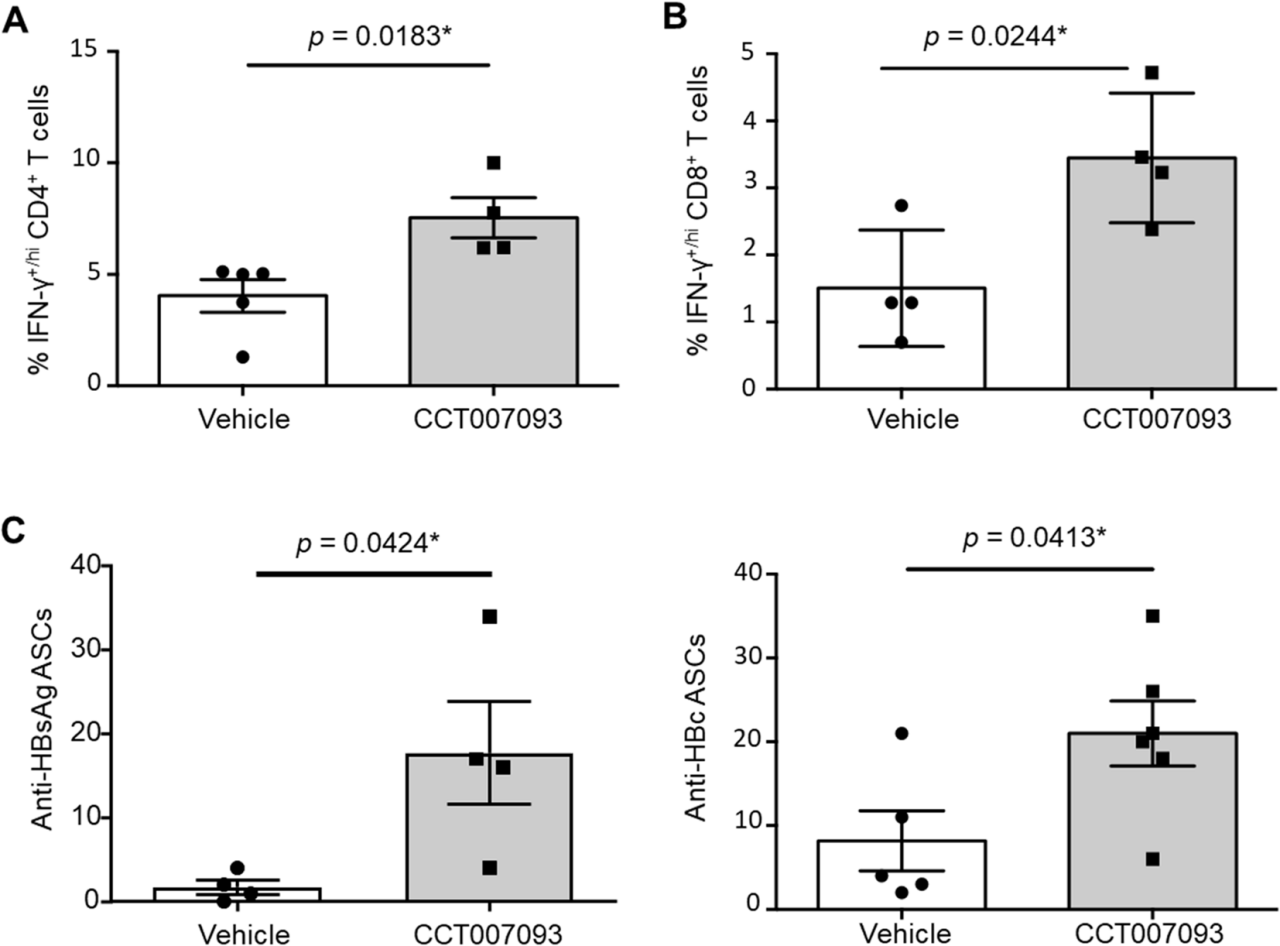
*Ex vivo* enhanced cytotoxicity and Ab production of intrahepatic lymphocytes from CCT007093 treated HBV-infected mice. Quantification of flow cytometric analysis showing the percentage of intrahepatic IFN-γ-producing T cells, including **(A)** CD4^+^ (n=4-5) and **(B)** CD8^+^ (n=4) T cells, isolated from mice treated with either vehicle or CCT007093. The T cells were subjected to ex vivo stimulation for 3 hr. Additionally, **(C)** The percentages of anti-HBsAg (left, n=4) and anti-HBcAg (right, n=5-6) antibody-secreting cells (ASCs) were quantified using ELISPOT assays and depicted in bar graphs to compare between mice treated with vehicle or CCT007093 during immune exhaustion induced by HBV infection. Statistical significance was determined using a two-tailed unpaired *t*-test, with significance levels indicated as follows: *P < 0.05.

Given that the increased expression of inhibitory receptors can lead to compromised functionality, including diminished IFN-γ production and reduced plasma cell (PC) numbers (21–23), we investigated whether the downregulation of PD-1 and FcγRIIB expression in intrahepatic lymphocytes following CCT007093 treatment could enhance IFN-γ production in T cells and boost the synthesis of antiviral Abs by B cells. To explore this, we cultured purified intrahepatic leukocytes from chronic HBV-infected mice and stimulated them with PMA and ionomycin for 3 hr. Following stimulation, we performed permeabilization and staining T cells using anti-IFN-γ Abs. As a result, we observed an increased frequency of IFN-γ^+/hi^CD4^+^ T cells (**Figure 5A**) and IFN-γ^+/hi^CD8^+^ T cells (**Figure 5B**) in the CCT007093-treated group. These findings shed light on the potential of CCT007093 to enhance the cytotoxicity of T cells in the liver, potentially ameliorating immune exhaustion in chronic HBV-infected mice.

While T cells play a central role in HBV clearance, the contribution of B cells should not be overlooked, given their ability to generate neutralizing antibodies (Abs) that provide an Ab-dependent shield against infection (24–26). In addition to investigating T-cell responses, we examined B-cell function to determine whether CCT007093-induced reduction in FcγRIIB expression within intrahepatic B cells influences PC production. To assess Ab-secreting cells (ASCs), we performed ELISPOT assays. As shown in **Figure 5C**, CCT007093-treated mice exhibited a significant increase in the numbers of HBsAg- and HBcAg-specific ASCs, indicating an enhanced humoral immune response in the liver. These findings suggest that CCT007093 promotes antiviral B-cell responses, complementing its effects on T-cell immunity.

### Down-regulation of transcriptional activities of PD-1 and FcγRIIB genes by CCT007093 treatment

**Figure 6:**
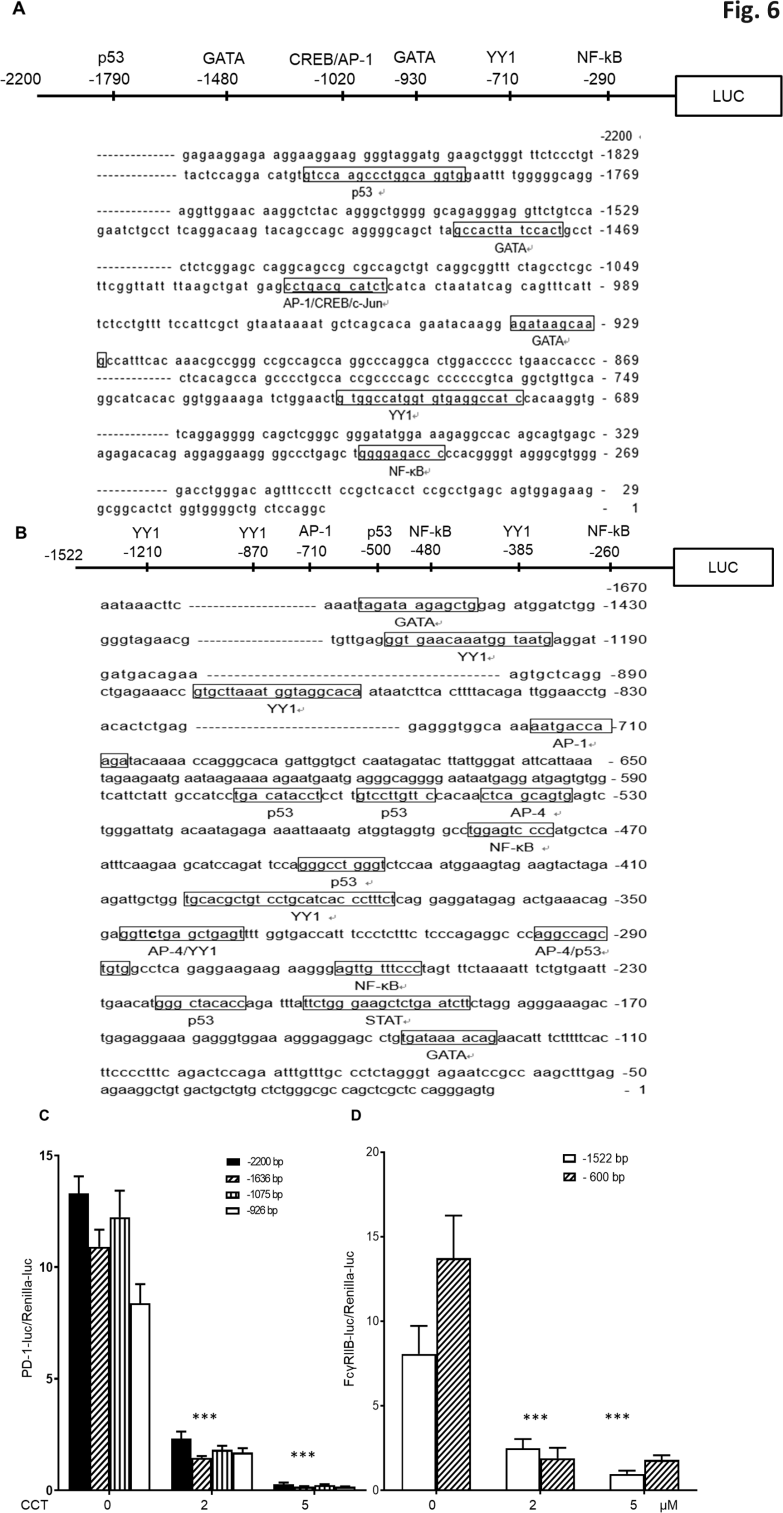
Reduction of PD-1 and FcγRIIB promoter transcriptional activities by CCT007093 treatment in BJAB B cells. **(A)** Illustration of predicted transcription factor binding sites within the –2,200 bp of the PD-1 promoter and **(B)** the –1,522 bp of FcγRIIB promoter employing TRANSFAC. **(C)** BJAB B cells (1-2×10^6^) were transiently co-transfected with either the –926 bp, –1075 bp, –1636 bp or –2200 bp promoter sequences of PD-1 pGL3 and renilla luciferase plasmids (pRL-TK). This transfection was conducted in the absence or presence of 2 or 5 μM CCT007093 for 24 hr to assess their promoter activities. Comparisons of each corresponding promoter sequences were significantly lower in response to 2 and 5 μM CCT007093 than vehicle (DMSO). **(D)** BJAB B cells were transiently co-transfected with either the –1522 bp or the –600 bp promoter sequences of FcγRIIB pGL3 and renilla pRL-TK plasmids in the absence or presence of 2 or 5 μM CCT007093 to evaluate the promoter activities linked to FcγRIIB. Comparisons of each corresponding promoter sequences were significantly lower in response to 2 and 5 μM CCT007093 than vehicle (DMSO). Statistical significance was determined using a two-tailed unpaired *t*-test, with significance levels indicated as follows: ***P < 0.001.

To further investigate the inhibitory effects of CCT007093 on Wip1 and its potential substrates, such as p53 and NF-κB, recognized as transcription factors (12), we performed a comprehensive promoter analysis to investigate the transcriptional regulation of PD-1 and FcγRIIB genes. Our analysis of the 2.2 kb promoter region upstream of transcriptional start site of the PD-1 gene identified potential transcriptional regulators, including p53, GATA, cAMP response element-binding protein (CREB), activator protein 1 (AP-1), Yin Yang 1 (YY1) and NF-κB (**Figure 6A**). Similarly, analysis of the 1.5 kb promoter region upstream of the transcriptional start site of the FcγRIIB gene revealed transcriptional regulators such as such as p53, AP-1, YY1 and NF-κB (**Figure 6B**).

Using this information, we conducted luciferase reporter gene assays to assess the transcriptional activity of these promoters. We generated various 5’ deletion mutants of the PD-1 and FcγRIIB gene promoters and inserted them into a firefly luciferase reporter construct for subsequent transfection experiments. The transcriptional activities of the PD-1 gene’s –2,200 bp, –1,636 bp, –1,075 bp, and –936 bp promoter constructs, as well as the FcγRIIB gene’s –1,522 bp, and –600 bp promoter constructs in BJAB B cells, both in the absence or presence of 2 or 5 μM of CCT007093 for 24 hr. The combined results indicated that the shortest promoters, specifically the –936 bp of the PD-1 gene and the –600 bp of FcγRIIB gene, retained their susceptibility to CCT007093 inhibition (**Figure 6C**). Comparative analysis of these two promoter regions highlighted the co-existence of YY1 and NF-κB, suggesting their potential roles as critical co-transcriptional regulators in the context of CCT007093 response.

### Effects of CCT007093 on the phosphorylation of transcriptional regulator substrates of Wip1

Given the concurrent down-regulation of PD-1 and FcγRIIB expression in T cells and B cells following CCT007093 treatment *in vivo* (**Figures 2**-**4**), it seems plausible to hypothesize the existence of co-regulators governing the transcription of these two genes. Our promoter analysis of the PD-1 and FcγRIIB genes suggests primary candidates for such transcriptional co-regulators are YY1 and NF-κB (**Figure 6**).

**Figure 7:**
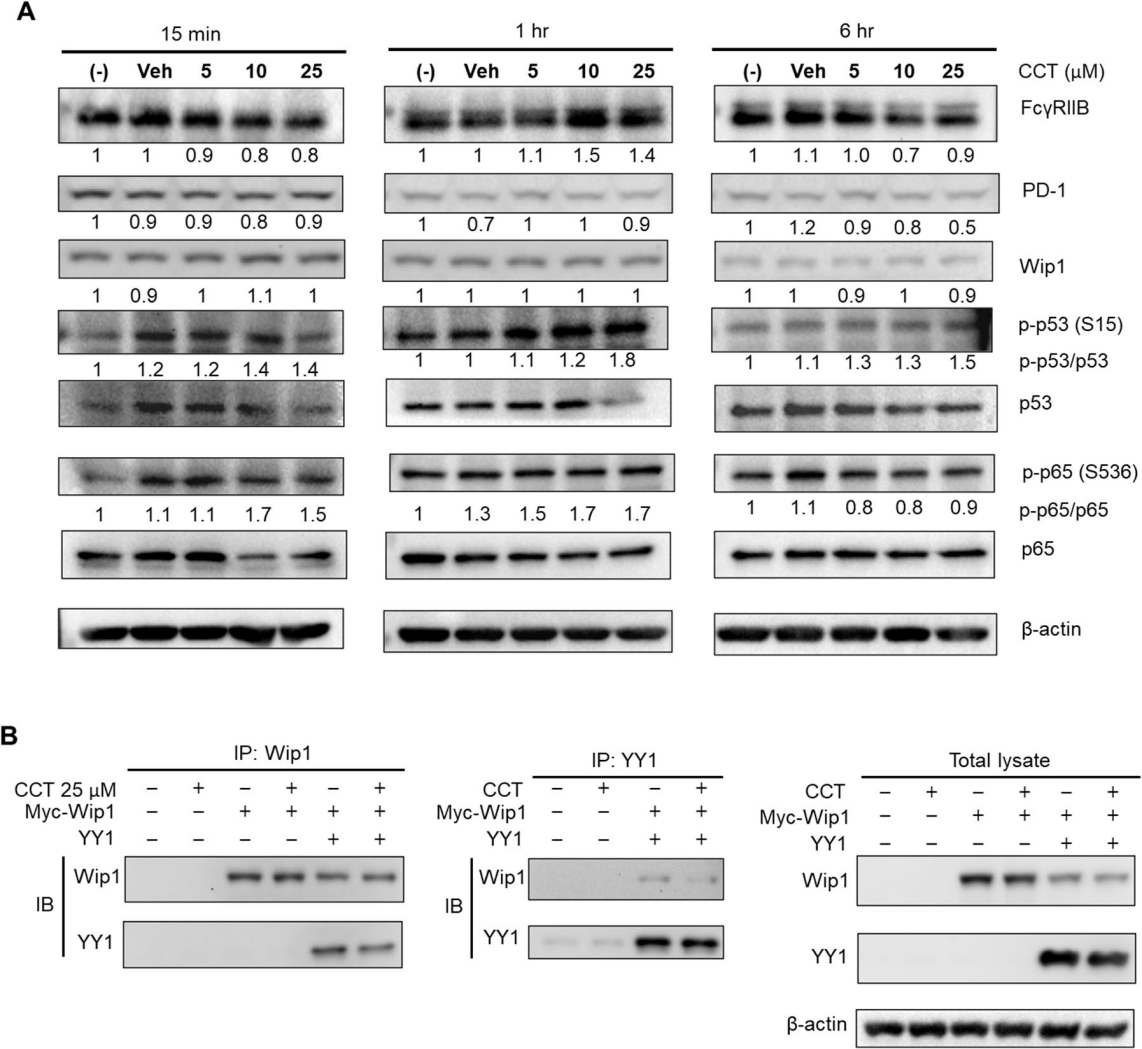
Impact of CCT007093 treatment on transcriptional regulators of PD-1 and FcγRIIB genes. **(A)** BJAB B cells (1×10^6^) were subjected to treatment with CCT007093 at concentrations of 0, 5, 10, or 25 μM for durations of 15 min, 1 hr, and 6 hr, respectively. After treatment, cells were harvested and lysed for western blotting, aiming to quantify the levels of PD-1, FcγRIIB, Wip1, p53, p-p53 (S15), p65 NF-κB, p-p65 (S536), and β-actin. As a standard for quantification, β-actin was employed as an internal control. **(B)** HEK293T cells (2.4×10^5^/ml) were transfected with myc-tagged Wip1 constructs, both with or without YY1 constructs. Subsequent to 24 hr of transfection, the cells underwent treatment with 25 μM CCT007093 for 1 hr, followed by harvesting for cell lysates. For immunoprecipitation, cell lysates were utilized with anti-Wip1 or anti-YY1 Abs, and immunoblotting was carried out utilizing the designated Abs.

Next, we aimed to investigate the effects of CCT007093 treatment on BJAB B cells using western blot analysis. According to previous studies, Wip1 targets the catalytic sites of p65 NF-κB and p53 at serine 535 and serine 15, respectively, thereby modulating their activity (12). To explore this, we treated BJAB B cells with increasing concentrations of CCT007093 (0, 5, 10 or 25 μM) for 15 min, 1 hr and 6 hr, followed by western blot analysis to assess phosphorylation changes. As shown in **Figure 7A**, PD-1 and FcγRIIB protein levels notably decreased at 6 hr in a dose-dependent manner. Wip1 inhibition by CCT007093 led to an initial increase in p-p65 NF-κB (Ser 535) phosphorylation at 15 min, although this effect was transient, reverting to basal phosphorylation levels by the 6-hour mark. On the contrary, p53 (Ser 15) phosphorylation was observed at 1 hr and persisted up to 6 hr. It is important to note that, despite the inhibition of Wip1’s phosphatase activity, the expression levels of Wip1 were unaffected by CCT007093 (**Figure 7A**).

Moreover, our study also suggests that YY1 might be a novel substrate of Wip1. We subsequently investigated if YY1 could directly interact with Wip1 within cells. As illustrated in **Figure 7B**, our co-transfection experiments in HEK293T cells showed a clear interaction between YY1 and Wip1, confirmed through co-immunoprecipitation. This finding implies that CCT007093 treatment may boost the phosphorylation of serine and/or threonine residues of YY1 by inhibiting Wip1.

### CCT007093-induced transcriptional regulatory pathways revealed by RNA-sequencing in T and B cells

**Figure 8:**
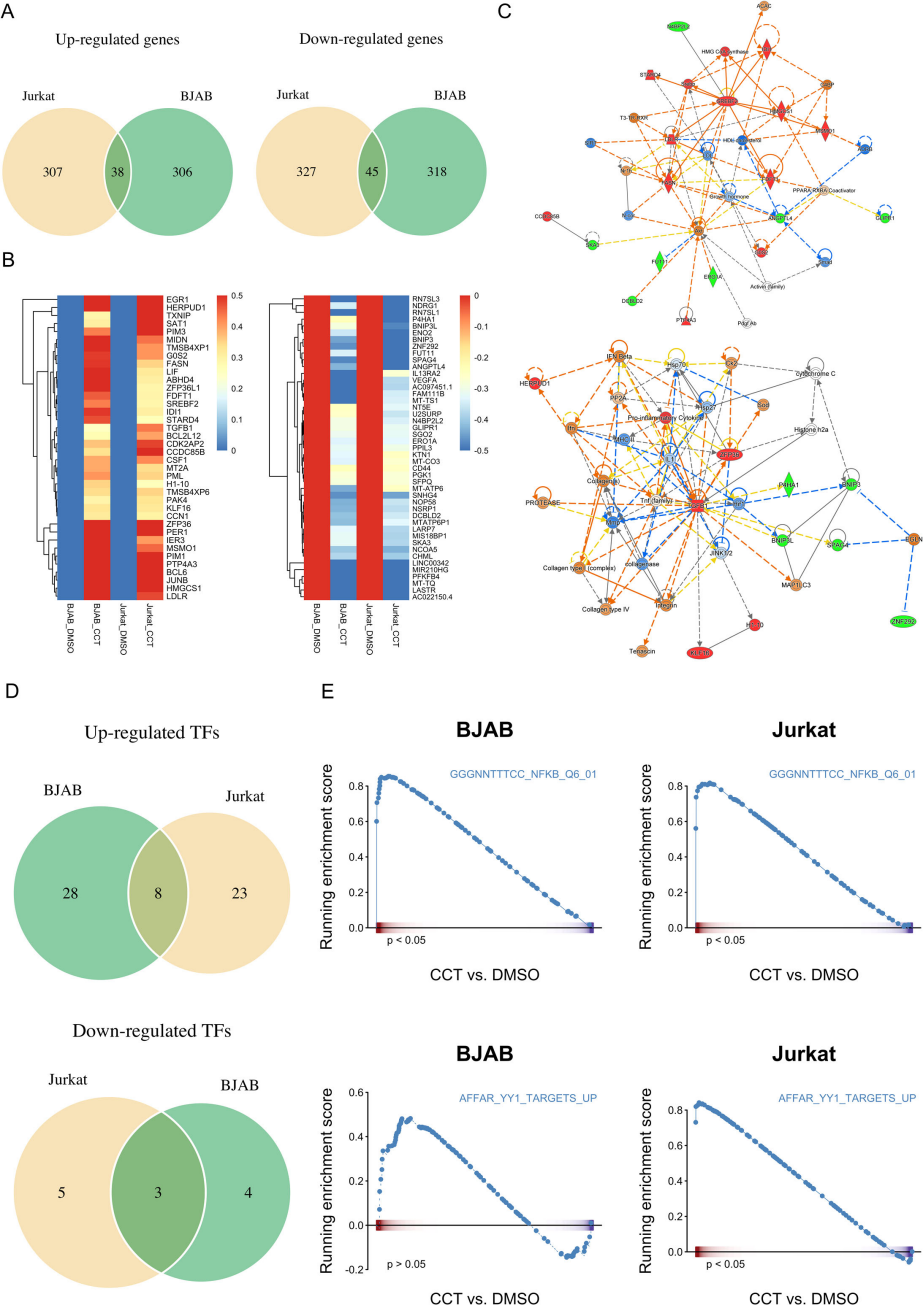
Comparative analysis of transcriptomics between vehicle and CCT007093 treated Jurkat T and BJAB B cells. Jurkat T cells and BJAB B cells (1×10^6^/ml) were treated with either vehicle or 25 μM CCT007093 for 16 hr. Following this, total RNAs were extracted for RNA-sequencing and data analysis. **(A)** Venn diagram illustrating the number of genes commonly up- (left) and down-regulated (right) in both Jurkat and BJAB cells. **(B)** A heatmap displaying the expression patterns of genes commonly up- and down-regulated in both Jurkat and BJAB cells. **(C)** Functional regulatory networks associated with inflammatory pathways were constructed based on genes commonly up- and down-regulated in both Jurkat and BJAB cells, analyzed using IPA. **(D)** Deciphering transcription factor (TF) activity in response to CCT007093 treatment. A Venn diagram was created to illustrate the number of TFs with commonly activated (top) and suppressed (bottom) transcriptional activity in both Jurkat and BJAB cells following CCT007093 treatment. **(E)** GSEA plots illustrate the expression of genes targeted by p65 NF-κB (top) and YY1 (bottom) in BJAB and Jurkat cells treated with CCT007093 compared to the vehicle. *P < 0.05 indicates statistical significance.

Given the identification of multiple substrates for Wip1 and the intricate network of transcription factors implicated in the regulation of PD-1 and FcγRIIB expression, as suggested by the promoter analysis and reporter gene assays (**Figure 7**), we performed bulk RNA-sequencing on Jurkat T cells and BJAB B cells. These cells were treated with either vehicle or 25 μM of CCT007093 for 16 hr. Whole transcriptomic sequencing and subsequent analyses revealed a set of shared up-regulated and down-regulated genes in both cell types (**Figures 8A, B**, **Supplementary Table 1**). Functional characterization of these genes through Gene Set Enrichment Analysis (GSEA) and Ingenuity Pathway Analysis (IPA) indicated their involvement in sterol metabolism pathways, such as the sterol regulatory binding protein (SREBP) pathway known to activate T cells and B cells (24, 27, 28), and cytokine-driven pro-inflammatory pathways activated by CCT007093 stimulation (**Figure 8C**). Notably, these up-regulated pathways are closely associated with activation of p65 NF-κB (29, 30), which had been earlier identified as a potential key transcription co-regulator in the promoter analysis of PD-1 and FcγRIIB genes (**Figure 8D**). Conversely, another key co-regulator, YY1, exhibited a positive correlation with the transcription factor-regulated pathway in Jurkat T cells (**Figure 8D**). Additional investigation through GSEA unveiled further potential transcriptional regulators, including TP53 and AP-1, which exhibited significant binding sites on the PD-1 and FcγRIIB promoters (**Figure 6**), activating their target genes in either Jurkat T cells, BJAB B cells, or both (**Supplementary Figure 9**, **Supplementary Table 2**).

## Discussion

Previous studies have demonstrated that PD-1/PD-L1 checkpoint blockades with blocking monoclonal Abs can restore antiviral intrahepatic T-cell responses in humans and in mice (7, 8). Based on this, we hypothesize that chemical compounds capable of down-regulating inhibitory receptors, such as PD-1 and FcγRIIB, may similarly enhance T and B cell activation. In this study we present compelling evidence that CCT007093 mitigates immune exhaustion in HBV-infected mice. Our results demonstrate that CCT007093 effectively reduce PD-1 and FcγRIIB expression across multiple compartments, including the peripheral blood, spleen, and liver (**Figures 1**-**4**). Furthermore, *ex vivo* analysis reveals enhanced T cell activation, characterized by increased IFN-γ production and heightened antiviral Ab secretion by B cells (**Figure 5**). Despite these promising effects on PD-1-expressing T cells, we found no concurrent changes of other inhibitory receptors, such as Tim-3, CTLA-4, and TIGIT, in exhausted T cells, suggesting a limitation of our HBV infection model (**Supplementary Figures 1**-**3**). Additionally, while CCT007093 appears to modulate PD-1 and PD-L1 to rejuvenate exhausted immune cells and promote viral clearance (**Figures 2**-**5**, **Supplementary Figure 4**), it may also exert direct effects on HBV-infected hepatocytes. Notably, previous studies indicate that CCT007093 enhances liver regeneration and improves survival following major hepatectomy (31), suggesting an additional indirect hepatoprotective role. Taken together, our findings support the concept that chemical checkpoint inhibition can effectively reactivate exhausted immune cells in chronic HBV infection in mice.

The mechanisms underlying CCT007093’s ability to inhibit Wip1, subsequently down-regulating PD-1 and FcγRIIB expression, are intricate, involving a network of regulatory pathways encompassing p53, p65 NF-κB, CREB, GATA, and YY1 (32). Considering Wip1’s known interaction with multiple transcription factors as its substrates (12), we conducted a comparative transcriptomic analysis between T cells and B cells. This exploration unveiled p65 NF-κB and YY1 as the central targets inhibited by Wip1, leading to the down-regulation of PD-1 and FcγRIIB, consequently promoting lymphocyte activation (**Figures 6**-**8**). These findings are consistent with the established role of activated p65 NF-κB in fostering the pro-inflammatory environment driving lymphocyte activation to bolster the adaptive immune response. Studies supporting our findings demonstrate that mice lacking Wip1 produce higher levels of pro-inflammatory cytokines–such as IL-1, IL-6, and IL-8–dependent on p65 NF-κB, in their splenic cells. Conversely, it has been evidenced that Wip1 overexpression can dose-dependently reduce p65 NF-κB activation *in vitro* (33). These findings suggest that Wip1 inhibition enhances lymphocyte activation by stimulating p65 NF-κB, which downregulates PD-1 and FcγRIIB expression, thereby lowering the activation threshold of lymphocytes and promoting further activation.

Beyond p65 NF-κB, previous research indicates that silencing YY1 can rejuvenate T cells in patients with chronic HIV infection (24). On the other hand, FcγRIIB has gained increasing recognition as an Ab checkpoint crucial for enhancing humoral protection (18, 34). Overexpression of YY1 has been shown to enhance the transcription of FcγRIIB gene in primary and transformed B cells. On the contrary, the loss of the single YY1 binding site at –385 region of promoter sequences (**Figure 6B**) due to a single nucleotide polymorphism significantly diminishes FcγRIIB expression (35). These observations suggest that YY1, rather than p65 NF-κB, primarily influences the transcriptional regulation of PD-1 and FcγRIIB. Therefore, silencing YY1 appears beneficial for the humoral response by promoting the generation of antiviral Abs for Ab-mediated cytotoxicity. However, PhosphoSitePlus (v6.7.1.1) notes that both mouse and human YY1 proteins contain numerous potential phosphorylation sites. Mouse YY1 has at least 8 serine residues and 4 threonine residues, while human YY1 has 10 serine residues and 3 threonine residues. Identifying the specific serine or threonine residue requiring phosphorylation to positively regulate the gene expression of PD-1 and FcγRIIB is crucial for the development of specific modulators.

The success of PD-1/PD-L1 checkpoint blockades has significantly expanded their applications in the treatment of cancer patients (7, 8). However, in the context of chronic infections and immune exhaustion, the application of anti-PD-1 and anti-PD-L1 therapies is notably less common. Despite their effectiveness, the high cost of PD-1/PD-L1 blockade therapy presents a substantial barrier to its utilization in chronic infections. Drawing from our findings, we propose the use of a chemical modulator for both PD-1 and FcγRIIB, e.g., CCT007093, to reactivate lymphocytes and enhance immune responses. When used in combination with antiviral drugs, this approach has the potential to significantly augment the efficacy of treatment for patients with chronic infections, especially those suffering from immune exhaustion. Compared to Ab-based PD-1/PD-L1 inhibitors, chemical drugs offer advantages in terms of affordability and safety due to their relatively short half life. Our findings from CCT007093 treatment also highlight several key transcription factors that could be targeted to reduce expression levels of PD-1 and FcγRIIB, either individually or concurrently. Importantly, our data strongly support the concept of dual or multiple targeting to inhibitory receptors to activate both the cytotoxic and humoral arms of the immune system, potentially resulting in superior therapeutic synergism. The optimal strategy would involve the use of small molecules to block the action of immune checkpoint receptors, combining them with antiviral treatments, and identifying the novel, safer and more effective immunotherapeutic targets.

Additionally, our findings have implications for the application of chemical checkpoint inhibitors for the treatment of cancer patients. Based on our data, we reason that CCT007093 may not be an effective remedy for cancer. This speculation primarily stems from the well-established role of p65 NF-κB in promoting tumor growth (32). Therefore, it is not surprising to know that CCT007093 treatment has shown inconsistent therapeutic benefits in different types of cancers with wild-type p53 (36). Nevertheless, our findings suggest that YY1 could be a more suitable target for enhancing T cell cytotoxicity and inhibiting tumor growth. YY1 is a ubiquitously distributed transcription factor belonging to the GLI-Kruppel class of zinc finger proteins. Its role as a transcriptional repressor or activator varies depending on the cellular context (37). In cancer cells, YY1 modulates immune resistance by enhancing PD-L1 expression (38, 39) and promotes cancer metastasis. These findings highlight YY1 as a more promising target for chemical drugs to alleviate immune exhaustion caused by cancer cells. As mentioned earlier, blocking FcγRIIB can enhance the production of anti-tumor Abs, which is crucial for enhancing cancer immunotherapy (25).

**Figure 9:**
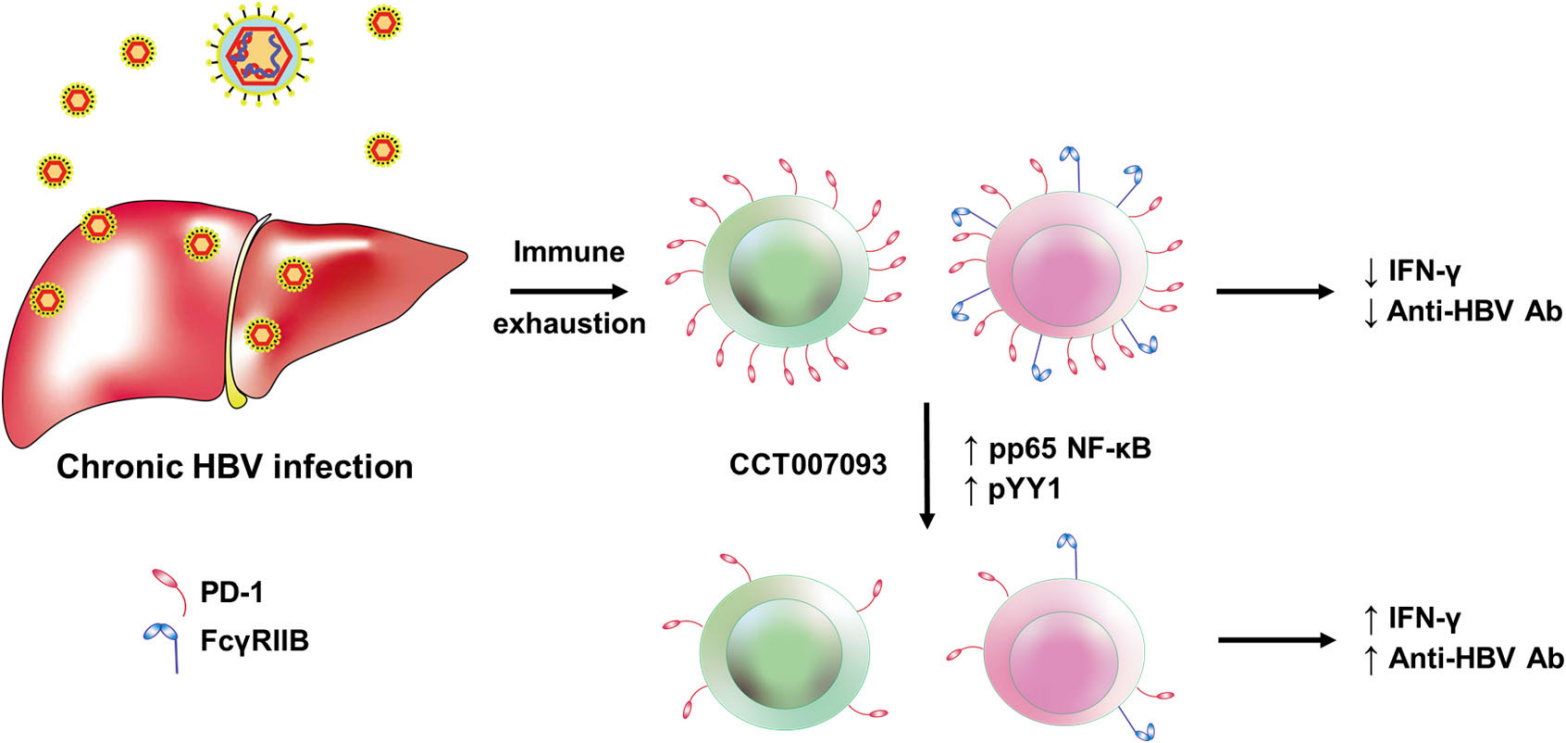
Graphic abstract on the CCT007093-mediated mechanisms in the amelioration of immune exhaustion induced by chronic HBV infection in mice. The mechanism of CCT007093 to rejuvenate immune cells involves p65 NF-κB and YY1 to modulate transcription of PD-1 and FcγRIIB genes.

In summary, we present a novel strategy to address immune exhaustion of lymphocytes and demonstrate its feasibility. CCT007093 and its derivatives emerge as promising candidate compounds for treating immune exhaustion in infectious diseases (**Figure 9**). Moreover, the exploration and characterization of chemical compounds to reduce the expression levels of inhibitory receptors hold significant potential for expanding therapeutic options in cancer patients. Furthermore, the critical role of FcγRIIB in enhancing humoral protection against infection and tumor appears to be under-recognized. Therefore, future research efforts should consider including FcγRIIB in the development of new checkpoint inhibitors. This is particularly important when safeguarding against acute bacterial and viral infections, as we and other researchers have recently affirmed (19, 40). Lastly, we sincerely hope our findings will serve as a stepping stone to inspire further research, particularly among clinicians, to advance investigations into immune exhaustion induced by chronic viral infections.

## Data Availability

The datasets presented in this study can be found in online repositories. The names of the repository/repositories and accession number(s) can be found in the article/**Supplementary Material**.

## Supplementary material

Supplementary Table 1 Differentially expressed genes in Jurkat and BJAB cells in response to vehicle or CCT007093. This supplementary table provides a list of genes that exhibit differential expression in the comparisons between vehicle (DMSO) versus CCT007093 treated Jurkat T and BJAB B cells. These gene lists were employed to construct the Venn diagram shown in **Figure 8A**. Log2FC denotes log2-transformed fold-change values.

Supplementary Table 2 Transcription factor targets showing enrichment in response to CCT007093 treatment in Jurkat or BJAB cells. The list of transcription factor targets was sourced from MSigDB’s c7-tft category, and the enrichment analysis was conducted using GSEA.

Supplementary Figure 1 Gating strategies for flow cytometric analysis of PD-1-expressing **(A)** CD4^+^ and CD8^+^ T cells, and **(B)** PD-1- and FcγRIIB-expressing B cells in the peripheral blood of vehicle- and CCT007093-treated mice.

Supplementary Figure 2 Gating strategies for flow cytometric analysis of PD-1-expressing **(A)** CD4^+^ and CD8^+^ T cells, **(B)** CD4^+^CD127^low^ and CD8^+^CD127^low^ T cells, and **(C)** PD-1- and FcγRIIB-expressing B cells in the spleen of vehicle- and CCT007093-treated mice.

Supplementary Figure 3 Gating strategies for flow cytometric analysis of **(A)** PD-1-expressing CD4^+^ and CD8^+^ T cells, **(B)** CD4^+^CD127^low^ and CD8^+^CD127^low^ T cells, and **(C)** PD-1- and FcγRIIB-expressing B cells in the liver of vehicle- and CCT007093-treated mice.

Supplementary Figure 4 Gating strategies for flow cytometric analysis of *ex vivo* IFN-γ-expressing CD4^+^ (Right bottom three plots) and CD8^+^ (Left bottom three plots) T cells isolated from the liver of vehicle- and CCT007093-treated mice. Fluorescence-minus-one (FMO) and isotype controls were included to accurately determine intracellular IFN-γ (+) T cells.

Supplementary Figure 5 Effects on CCT007093 on the expression of Tim-3, CTLA-4 and TIGIT in circulating T cells of chronic HBV-infected mice. **(A)** Flow cytometry analysis of CD4^+^ T cells in peripheral blood. (Left panels) Percentages of Tim-3^+^ (top), CTLA-4^+^ (middle), and TIGIT^+^ (bottom) CD4^+^ T cells. (Left panels) Corresponding mean fluorescence intensity (MFI) levels of these markers in CD4^+^ T cells from mice treated with either vehicle (white bars, solid circles; n=6-9) or CCT007093 (gray bars, solid squares; n=10–12). **(B)** Flow cytometry analysis of CD8^+^ T cells in the peripheral blood. (Right panels) Percentages of Tim-3^+^ (top), CTLA-4^+^ (middle), and TIGIT^+^ (bottom) CD8^+^ T cells. (Right panels) Corresponding MFI levels of these markers in CD8^+^ T cells from mice treated with either vehicle (white bars, solid circles; n=5-9) or CCT007093 (gray bars, solid squares; n=6–10). No statistical significance was observed in any of the indicated comparisons using the two-tailed unpaired *t*-test.

Supplementary Figure 6 Effects of CCT007093 on the expression of Tim-3, CTLA-4, and TIGIT in splenic T cells of chronic HBV-infected mice. **(A)** Flow cytometry analysis of CD4^+^ T cells in the spleen. (Left panels) Percentages of Tim-3^+^ (top), CTLA-4^+^ (middle), and TIGIT^+^ (bottom) CD4^+^ T cells. (Right panels) Corresponding MFI levels of these markers in CD4^+^ T cells from mice treated with either vehicle (white bars, solid circles; n=6–9) or CCT007093 (gray bars, solid squares; n=10–12). **(B)** Flow cytometry analysis of CD8^+^ T cells in the spleen. (Right panels) Percentages of Tim-3^+^ (top), CTLA-4^+^ (middle), and TIGIT^+^ (bottom) CD8^+^ T cells. (Right panels) Corresponding MFI levels of these markers in CD8^+^ T cells from mice treated with either vehicle (white bars, solid circles; n=5–9) or CCT007093 (gray bars, solid squares; n=6–10). No statistically significant differences were observed in any of the comparisons, as determined by a two-tailed unpaired *t*-test.

Supplementary Figure 7 Effects of CCT007093 on the expression of Tim-3, CTLA-4, and TIGIT in intrahepatic T cells of chronic HBV-infected mice. **(A)** Flow cytometry analysis of CD4^+^ T cells in the liver. (Left panels) Percentages of Tim-3^+^ (top), CTLA-4^+^ (middle), and TIGIT^+^ (bottom) CD4^+^ T cells. (Right panels) Corresponding MFI levels of these markers in CD4^+^ T cells from mice treated with either vehicle (white bars, solid circles; n=6–9) or CCT007093 (gray bars, solid squares; n=10–12). **(B)** Flow cytometry analysis of CD8^+^ T cells in the liver. (Left panels) Percentages of Tim-3^+^ (top), CTLA-4^+^ (middle), and TIGIT^+^ (bottom) CD8^+^ T cells. (Right panels) Corresponding MFI levels of these markers in CD8^+^ T cells from mice treated with either vehicle (white bars, solid circles; n=5–9) or CCT007093 (gray bars, solid squares; n=6–10). No statistically significant differences were observed in any comparisons, as determined by a two-tailed unpaired *t*-test.

Supplementary Figure 8 Effects of CCT007093 on PD-L1 expression in hepatic Kupffer cells of chronic HBV-infected mice. **(A)** Representative flow cytometric plots showing hepatic Kupffer cells (CD11b^+^F4/80^+/hi^, highlighted in red boxes) gated from CD45^+^FSC^+^ populations, followed by CD3^-^CD11b^+^ gating, in mice treated with either vehicle (left) or CCT007093 (right). **(B)** Bar graphs showing the percentages (left) of Kupffer cells in CD11b^+^ myeloid cells and their corresponding PD-L1 MFI levels (right) in mice treated with either vehicle (white bars, solid circles; n=5) or CCT007093 (gray bars, solid squares; n=6). Statistical significance was determined using a two-tailed unpaired *t*-test, with significance levels indicated as follows: **P* < 0.05, ****P* < 0.001.

Supplementary Figure 9 GSEA plots of genes targeted by TFs in cells treated with CCT007093 compared to vehicle. List of TFs showing significant differences in GSEA plots between cells treated with vehicle versus CCT007093. GSEA plots illustrating the expression of genes targeted by relevant (**Figures 6**, **8**) and significant TFs in Jurkat T and BJAB B cells treated with CCT007093 compared to vehicle. The list includes: **(A)** Tumor protein 53 (TP53), **(B)** Cyclic AMP-responsive element-binding protein 1 (CREB1), **(C)** Androgen receptor (AR), **(D)** Zinc finger protein 589 (ZNF589), **(E)** Nuclear factor Y (NF-Y, also known as CCAAT-binding factor (CBF)), **(F)** Thyroid hormone receptor α (THRA), **(G)** Activator protein 1 (AP-1), **(H)** Mitogen-activated protein kinase 3 (MAPK3, also known as p44 MAPK and ERK1), **(I)** Serum response factor (SRF), **(J)** General transcription factor IIE subunit 2 (GTF2E2), and **(K)** Double-stranded RNA-specific endoribonuclease (DROSHA) genes. *P* < 0.05 indicates statistical significance.
