# Supplementary figures and images for: Wip1 inhibitor CCT007093 alleviates immune exhaustion of lymphocytes via p65 NF-κB and YY1 in chronic hepatitis B virus infection in mice
Source: Front Immunol. 2025 May 9;16:1548814. doi: 10.3389/fimmu.2025.1548814 (PMC12098592; doi:10.3389/fimmu.2025.1548814)

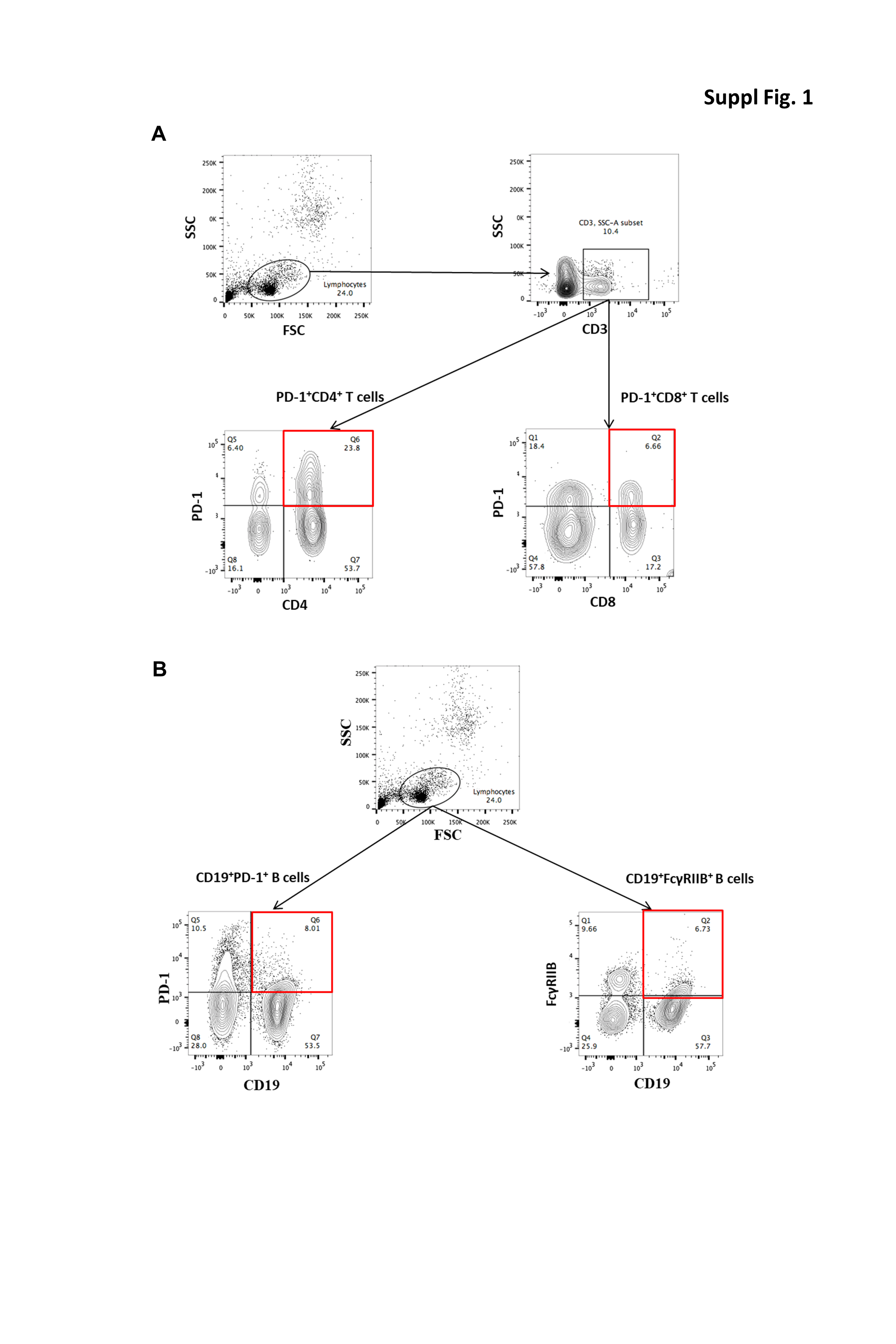

Supplement: Supplementary Figure 1 — Gating strategies for flow cytometric analysis of PD-1-expressing (A) CD4+ and CD8+ T cells, and (B) PD-1- and FcγRIIB-expressing B cells in the peripheral blood of vehicle- and CCT007093-treated mice. [file Image1.tif]

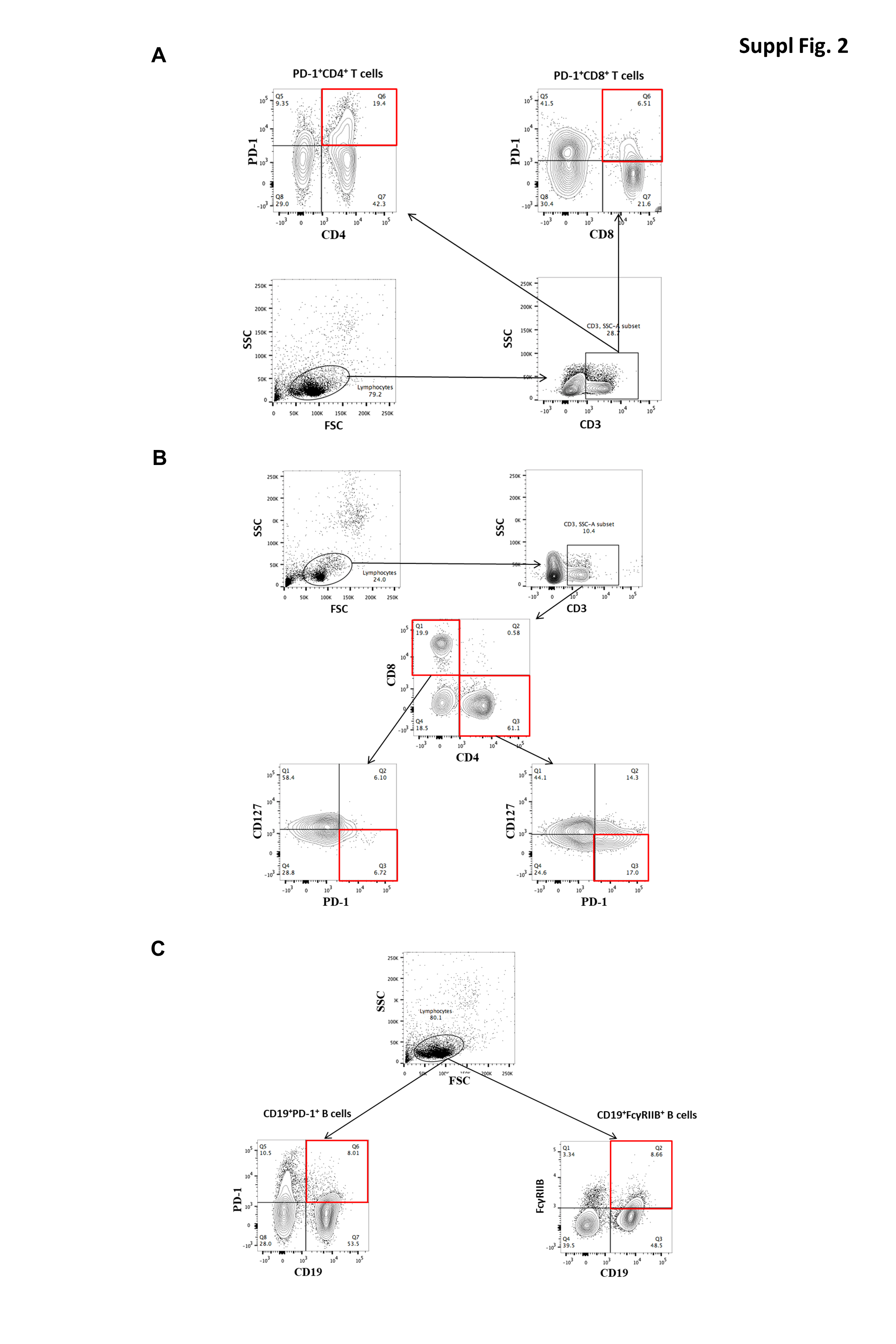

Supplement: Supplementary Figure 2 — Gating strategies for flow cytometric analysis of PD-1-expressing (A) CD4+ and CD8+ T cells, (B) CD4+CD127low and CD8+CD127low T cells, and (C) PD-1- and FcγRIIB-expressing B cells in the spleen of vehicle- and CCT007093-treated mice. [file Image2.tif]

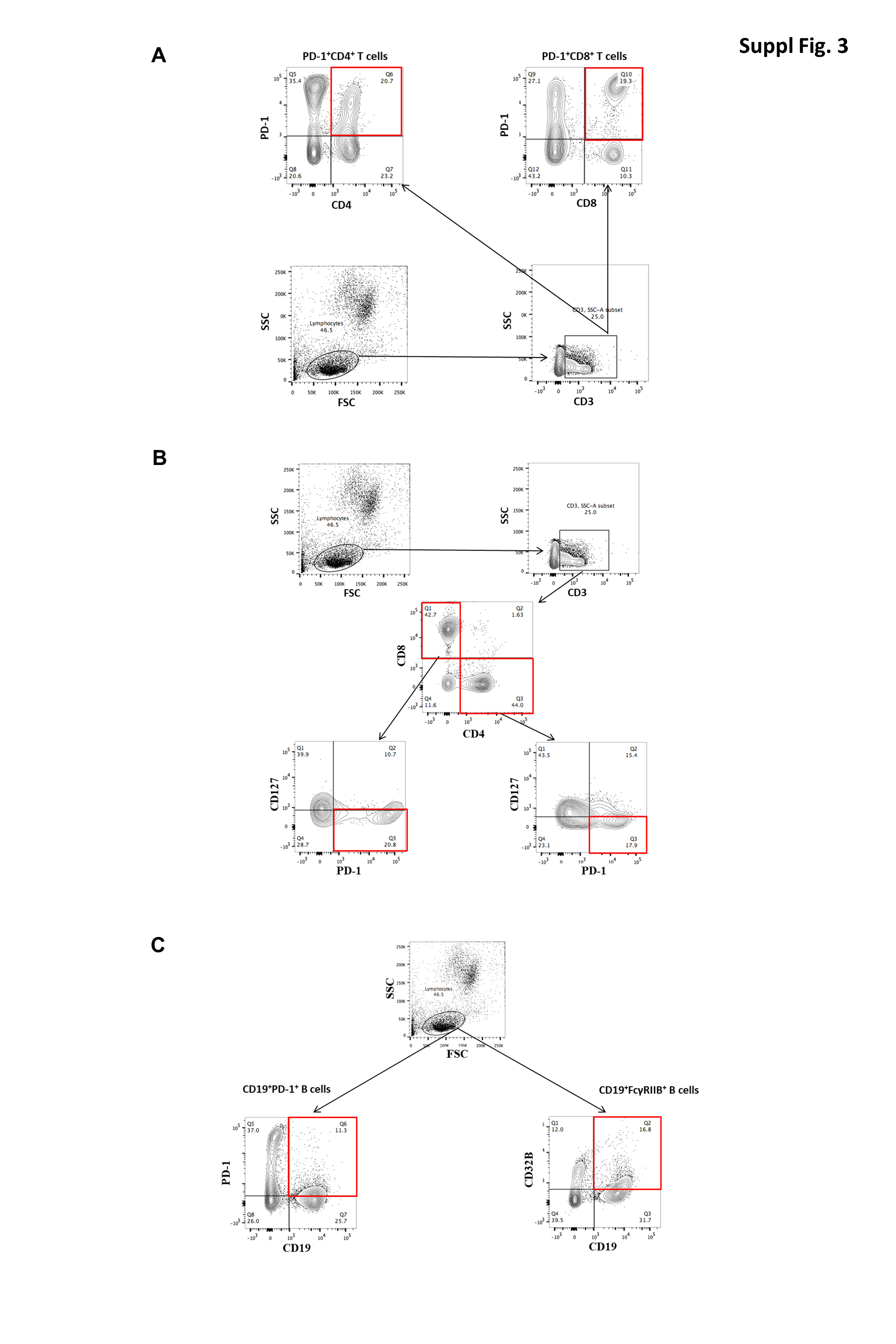

Supplement: Supplementary Figure 3 — Gating strategies for flow cytometric analysis of (A) PD-1-expressing CD4+ and CD8+ T cells, (B) CD4+CD127low and CD8+CD127low T cells, and (C) PD-1- and FcγRIIB-expressing B cells in the liver of vehicle- and CCT007093-treated mice. [file Image3.tif]

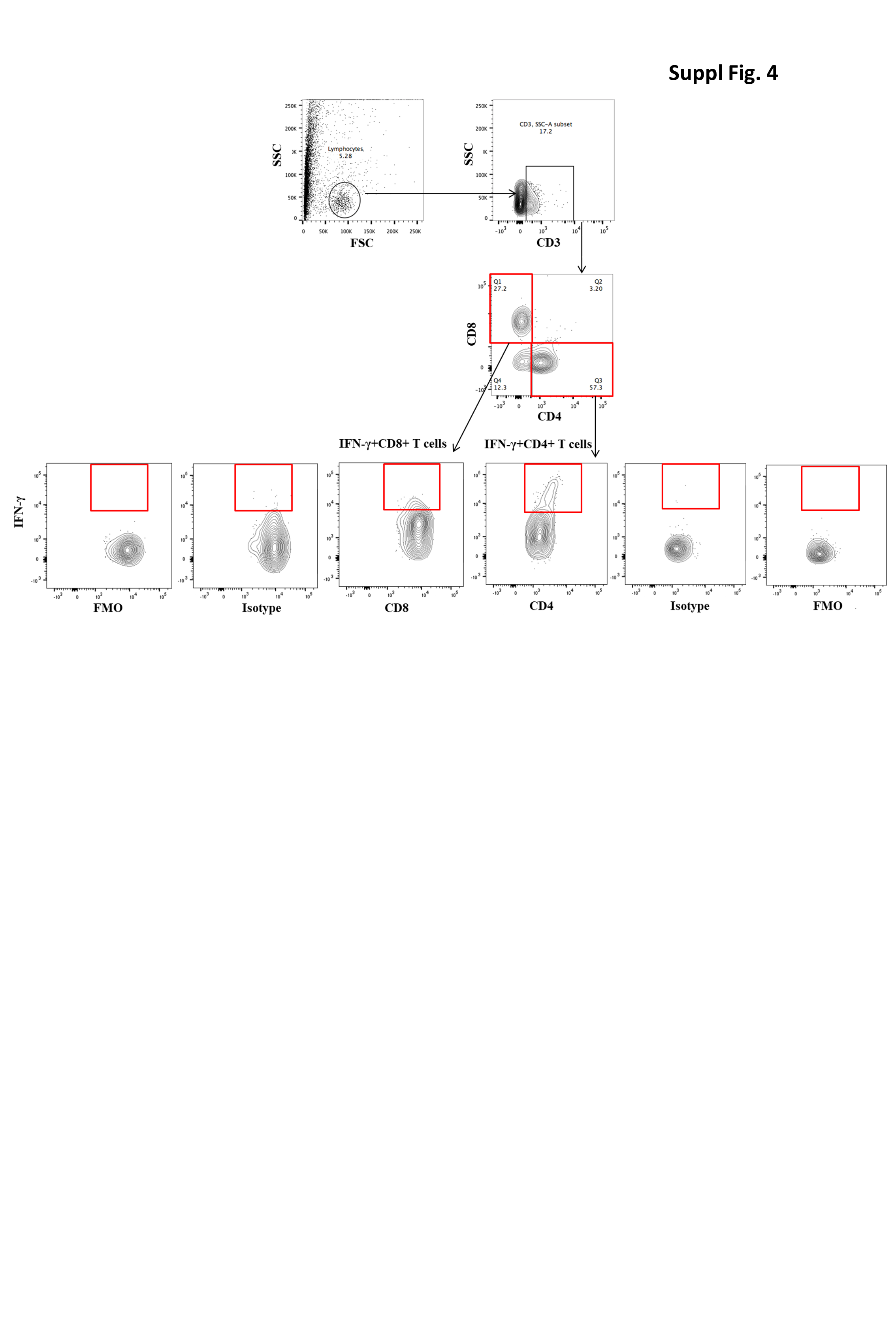

Supplement: Supplementary Figure 4 — Gating strategies for flow cytometric analysis of ex vivo IFN-γ-expressing CD4+ (Right bottom three plots) and CD8+ (Left bottom three plots) T cells isolated from the liver of vehicle- and CCT007093-treated mice. Fluorescence-minus-one (FMO) and isotype controls were included to accurately determine intracellular IFN-γ (+) T cells. [file Image4.tif]

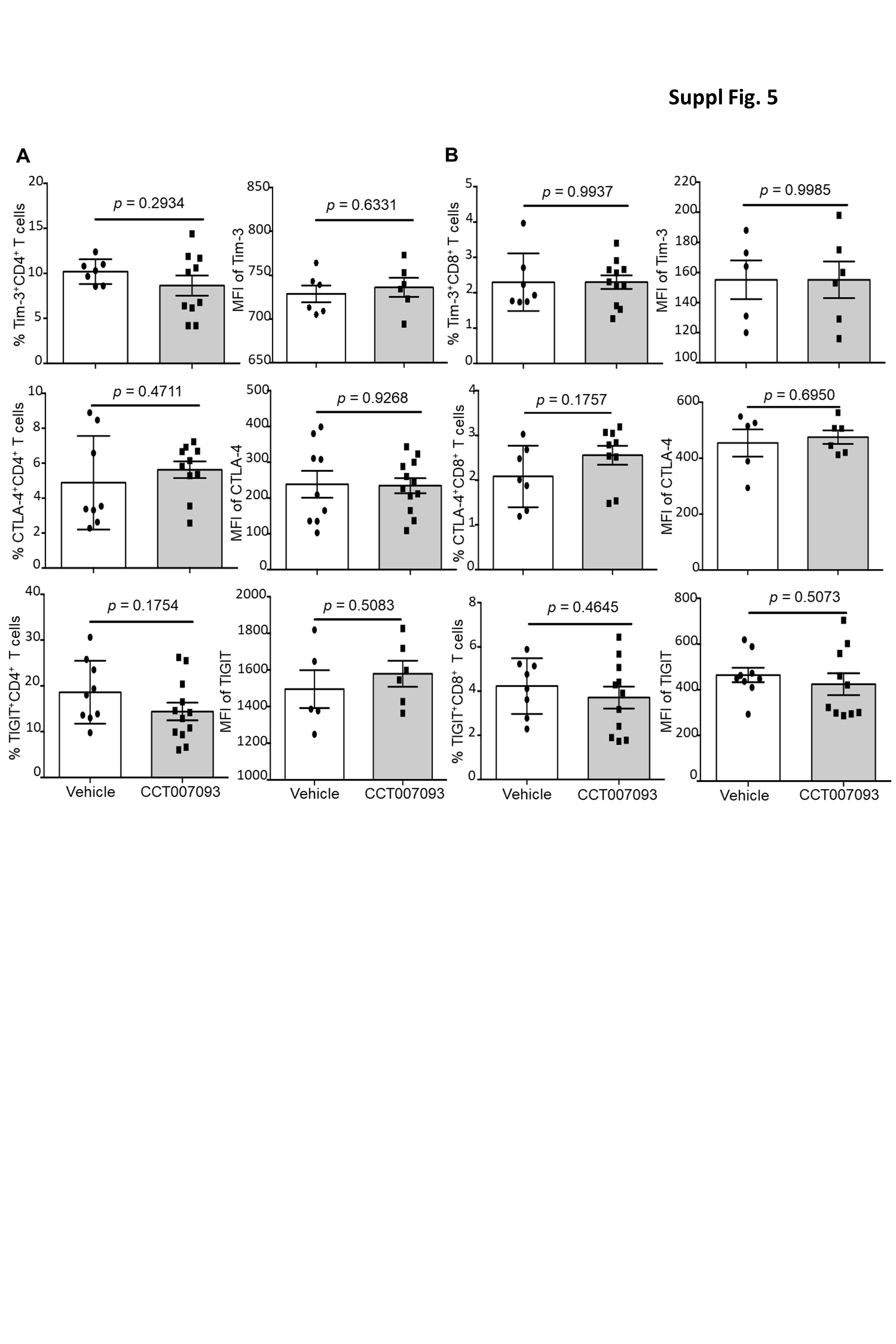

Supplement: Supplementary Figure 5 — Effects on CCT007093 on the expression of Tim-3, CTLA-4 and TIGIT in circulating T cells of chronic HBV-infected mice. (A) Flow cytometry analysis of CD4+ T cells in peripheral blood. (Left panels) Percentages of Tim-3+ (top), CTLA-4+ (middle), and TIGIT+ (bottom) CD4+ T cells. (Left panels) Corresponding mean fluorescence intensity (MFI) levels of these markers in CD4+ T cells from mice treated with either vehicle (white bars, solid circles; n=6-9) or CCT007093 (gray bars, solid squares; n=10–12). (B) Flow cytometry analysis of CD8+ T cells in the peripheral blood. (Right panels) Percentages of Tim-3+ (top), CTLA-4+ (middle), and TIGIT+ (bottom) CD8+ T cells. (Right panels) Corresponding MFI levels of these markers in CD8+ T cells from mice treated with either vehicle (white bars, solid circles; n=5-9) or CCT007093 (gray bars, solid squares; n=6–10). No statistical significance was observed in any of the indicated comparisons using the two-tailed unpaired t-test. [file Image5.tif]

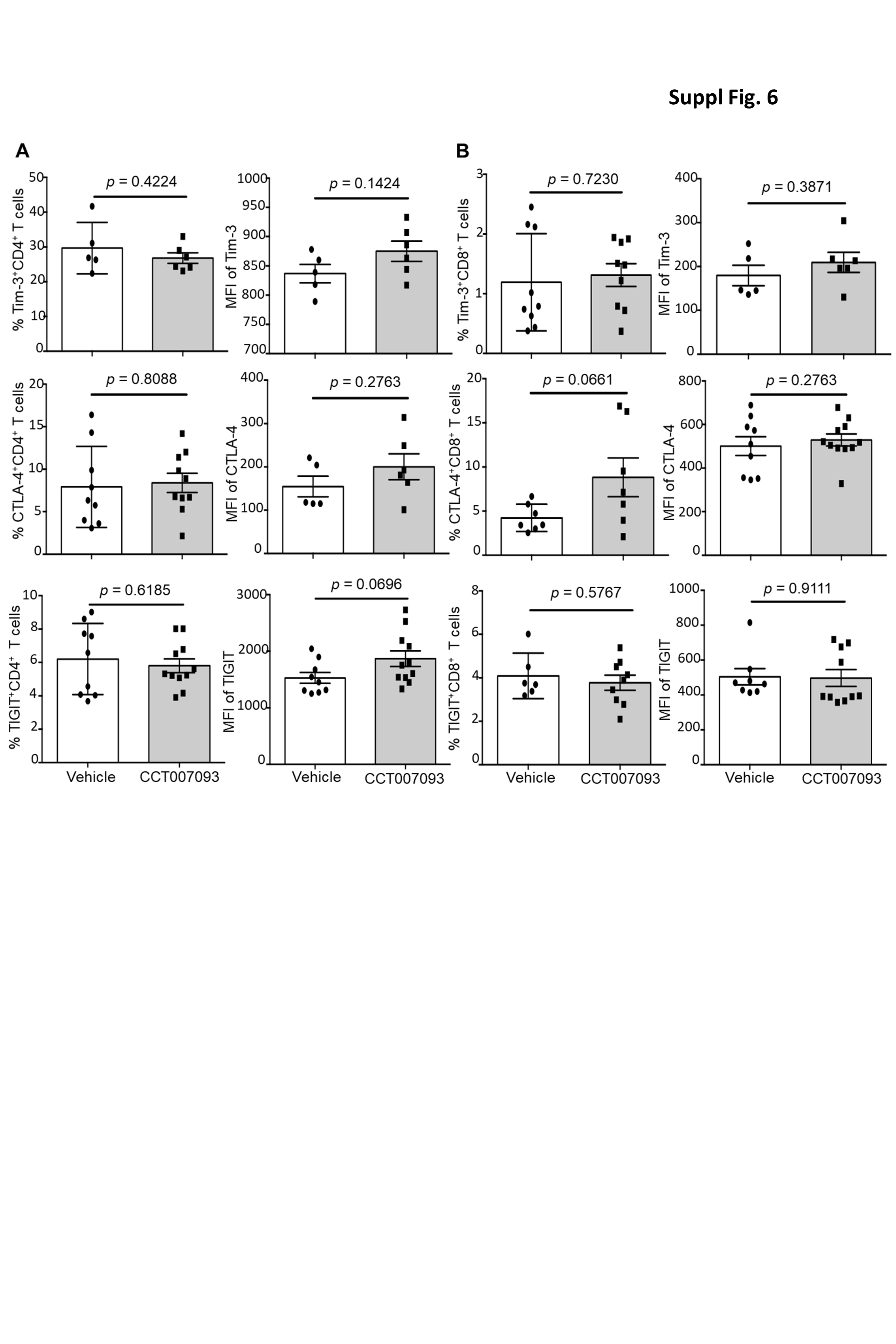

Supplement: Supplementary Figure 6 — Effects of CCT007093 on the expression of Tim-3, CTLA-4, and TIGIT in splenic T cells of chronic HBV-infected mice. (A) Flow cytometry analysis of CD4+ T cells in the spleen. (Left panels) Percentages of Tim-3+ (top), CTLA-4+ (middle), and TIGIT+ (bottom) CD4+ T cells. (Right panels) Corresponding MFI levels of these markers in CD4+ T cells from mice treated with either vehicle (white bars, solid circles; n=6–9) or CCT007093 (gray bars, solid squares; n=10–12). (B) Flow cytometry analysis of CD8+ T cells in the spleen. (Right panels) Percentages of Tim-3+ (top), CTLA-4+ (middle), and TIGIT+ (bottom) CD8+ T cells. (Right panels) Corresponding MFI levels of these markers in CD8+ T cells from mice treated with either vehicle (white bars, solid circles; n=5–9) or CCT007093 (gray bars, solid squares; n=6–10). No statistically significant differences were observed in any of the comparisons, as determined by a two-tailed unpaired t-test. [file Image6.tif]

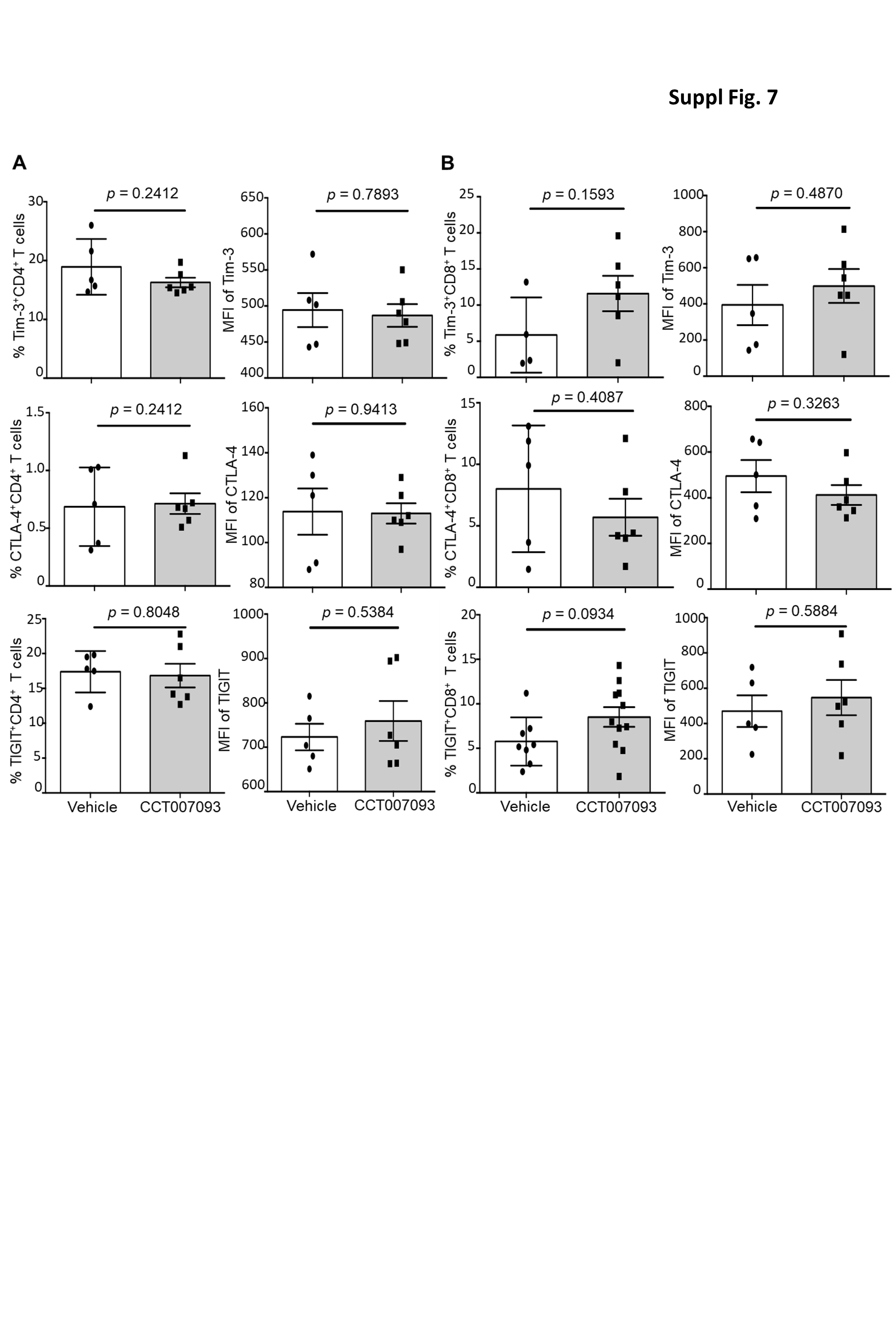

Supplement: Supplementary Figure 7 — Effects of CCT007093 on the expression of Tim-3, CTLA-4, and TIGIT in intrahepatic T cells of chronic HBV-infected mice. (A) Flow cytometry analysis of CD4+ T cells in the liver. (Left panels) Percentages of Tim-3+ (top), CTLA-4+ (middle), and TIGIT+ (bottom) CD4+ T cells. (Right panels) Corresponding MFI levels of these markers in CD4+ T cells from mice treated with either vehicle (white bars, solid circles; n=6–9) or CCT007093 (gray bars, solid squares; n=10–12). (B) Flow cytometry analysis of CD8+ T cells in the liver. (Left panels) Percentages of Tim-3+ (top), CTLA-4+ (middle), and TIGIT+ (bottom) CD8+ T cells. (Right panels) Corresponding MFI levels of these markers in CD8+ T cells from mice treated with either vehicle (white bars, solid circles; n=5–9) or CCT007093 (gray bars, solid squares; n=6–10). No statistically significant differences were observed in any comparisons, as determined by a two-tailed unpaired t-test. [file Image7.tif]

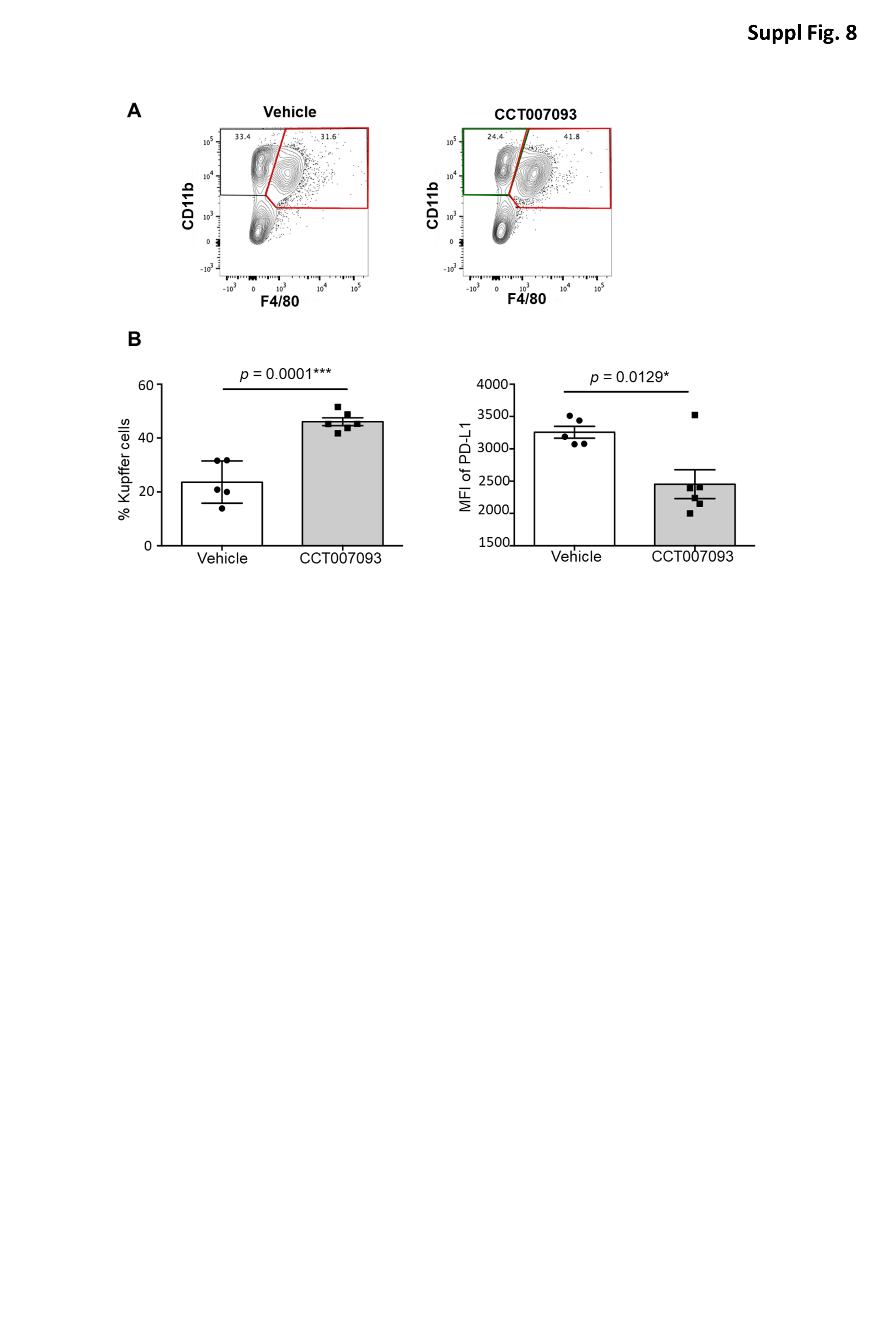

Supplement: Supplementary Figure 8 — Effects of CCT007093 on PD-L1 expression in hepatic Kupffer cells of chronic HBV-infected mice. (A) Representative flow cytometric plots showing hepatic Kupffer cells (CD11b+F4/80+/hi, highlighted in red boxes) gated from CD45+FSC+ populations, followed by CD3-CD11b+ gating, in mice treated with either vehicle (left) or CCT007093 (right). (B) Bar graphs showing the percentages (left) of Kupffer cells in CD11b+ myeloid cells and their corresponding PD-L1 MFI levels (right) in mice treated with either vehicle (white bars, solid circles; n=5) or CCT007093 (gray bars, solid squares; n=6). Statistical significance was determined using a two-tailed unpaired t-test, with significance levels indicated as follows: *P < 0.05, ***P < 0.001. [file Image8.tif]

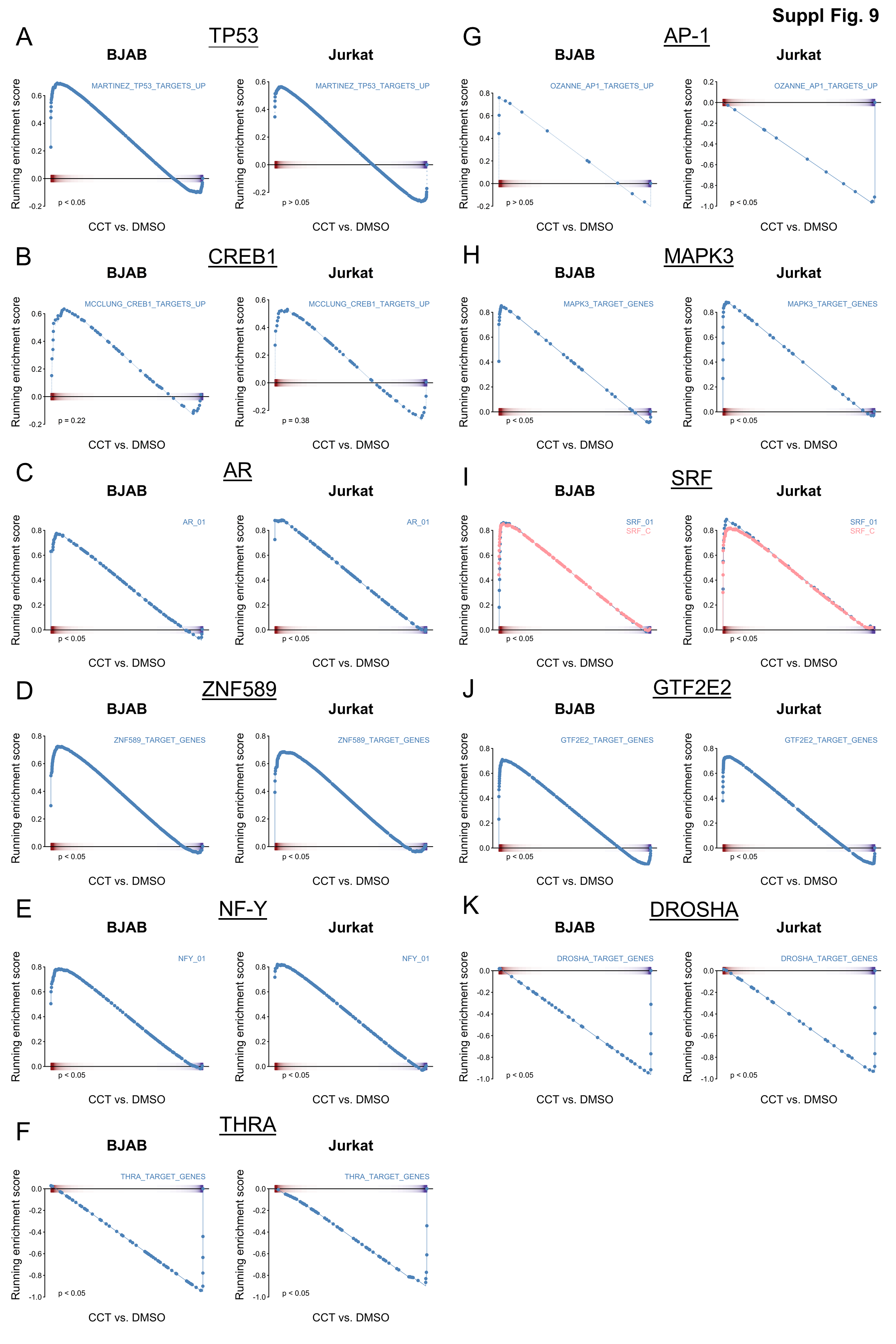

Supplement: Supplementary Figure 9 — GSEA plots of genes targeted by TFs in cells treated with CCT007093 compared to vehicle. List of TFs showing significant differences in GSEA plots between cells treated with vehicle versus CCT007093. GSEA plots illustrating the expression of genes targeted by relevant ( Figures 6 , 8 ) and significant TFs in Jurkat T and BJAB B cells treated with CCT007093 compared to vehicle. The list includes: (A) Tumor protein 53 (TP53), (B) Cyclic AMP-responsive element-binding protein 1 (CREB1), (C) Androgen receptor (AR), (D) Zinc finger protein 589 (ZNF589), (E) Nuclear factor Y (NF-Y, also known as CCAAT-binding factor (CBF)), (F) Thyroid hormone receptor α (THRA), (G) Activator protein 1 (AP-1), (H) Mitogen-activated protein kinase 3 (MAPK3, also known as p44 MAPK and ERK1), (I) Serum response factor (SRF), (J) General transcription factor IIE subunit 2 (GTF2E2), and (K) Double-stranded RNA-specific endoribonuclease (DROSHA) genes. P < 0.05 indicates statistical significance. [file Image9.tif]
